# Soy Peptide Supplementation Mitigates Undernutrition through Reprogramming Hepatic Metabolism in a Novel Undernourished Non‐Human Primate Model

**DOI:** 10.1002/advs.202306890

**Published:** 2024-05-30

**Authors:** Zhenzhen Xu, William Kwame Amakye, Zhengyu Ren, Yongzhao Xu, Wei Liu, Congcong Gong, Chiwai Wong, Li Gao, Zikuan Zhao, Min Wang, Tao Yan, Zhiming Ye, Jun Zhong, Chuanli Hou, Miao Zhao, Can Qiu, Jieqiong Tan, Xin Xu, Guoyan Liu, Maojin Yao, Jiaoyan Ren

**Affiliations:** ^1^ School of Food Science and Engineering South China University of Technology Guangzhou 510640 China; ^2^ The First Affiliated Hospital of Guangzhou Medical University Guangzhou Institute of Respiratory Disease & China State Key Laboratory of Respiratory Disease Guangzhou 510182 China; ^3^ State Key Laboratory of Quality Research in Chinese Medicine Institute of Chinese Medical Sciences (ICMS) University of Macau Macau 999078 China; ^4^ Huazhen Laboratory Animal Breeding Center Guangzhou 510900 China; ^5^ Center for Medical Genetics and Hunan Key Laboratory of Medical Genetics School of Life Science Central South University Changsha 410013 P. R. China; ^6^ College of Food Science and Engineering Yangzhou University Yangzhou 225127 China

**Keywords:** hepatic lipid metabolism, mitochondria dysfunction, non‐human primate, soypeptide, undernutrition

## Abstract

In spite of recent advances in the field of undernutrition, current dietary therapy relying on the supply of high protein high calorie formulas is still plagued with transient recovery of impaired organs resulting in significant relapse of cases. This is partly attributed to the inadequacy of current research models in recapitulating clinical undernutrition for mechanistic exploration. Using 1636 *Macaca fascicularis* monkeys, a human‐relevant criterion for determining undernutrition weight‐for‐age z‐score (WAZ), with a cutoff point of ≤ −1.83 is established as the benchmark for identifying undernourished nonhuman primates (U‐NHPs). In U‐NHPs, pathological anomalies in multi‐organs are revealed. In particular, severe dysregulation of hepatic lipid metabolism characterized by impaired fatty acid oxidation due to mitochondria dysfunction, but unlikely peroxisome disorder, is identified as the anchor metabolic aberration in U‐NHPs. Mitochondria dysfunction is typified by reduced mito‐number, accumulated long‐chain fatty acids, and disruption of OXPHOS complexes. Soy peptide‐treated U‐NHPs increase in WAZ scores, in addition to attenuated mitochondria dysfunction and restored OXPHOS complex levels. Herein, innovative criteria for identifying U‐NHPs are developed, and unknown molecular mechanisms of undernutrition are revealed hitherto, and it is further proved that soypeptide supplementation reprogramed mitochondrial function to re‐establish lipid metabolism balance and mitigated undernutrition.

## Graphical abstract

Using 1636 *Macaca fascicularis* monkeys, a human‐relevant criterion, WAZ, for determining undernutrition in NHPs is developed. WAZ ≤ −1.83 is established as the criteria for identifying undernourished NHPs (U‐NHPs). This novel model is then used to reveal hitherto unknown molecular mechanisms of undernutrition, and further proves that soy peptide supplementation reprogramed mitochondrial function to re‐establish lipid metabolism balance and mitigated undernutrition.

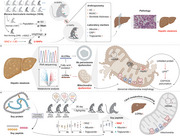

## 1 Introduction

Malnutrition is a deleterious health risk resulting from an imbalance between nutrient requirements and availability for normal physiological functioning. Broadly, this manifests as overnutrition, the excessive intake of calories, leading to disproportionate accumulation of fat and weight gain (obesity)^[^ ^1^ ^]^ or undernutrition, which is the insufficient intake of calories and essential nutrients, typified by stunted growth, weight loss, weakened immune system, and impaired physical and mental development.^[^ ^2^ ^]^ Undernutrition differs from cachexia, the wasting syndrome characterized by progressive weight loss, muscle wasting, and loss of body fat that is often associated with chronic illnesses, such as cancer, heart failure, chronic obstructive pulmonary disease (COPD), and certain autoimmune disorders.^[^ ^3^ ^]^ Globally, undernutrition poses not only severe health risk and increased morbidity and mortality,^[^ ^3^ ^]^ but also a great threat to economic development through loss of productivity.^[^ ^4^ ^]^ Unfortunately, this risk has been further exacerbated by pandemic outbreaks and protracted conflicts thereby making the need to surmount this universal health challenge, even more pressing.^[^ ^5^ ^]^ Severe undernutrition is mainly seen as extreme weight loss and complex phenotypical characteristics including dry skin and lesions, loss of hair pigmentation and edema caused by insufficient nutrient especially protein intake.^[^ ^3^, ^6^ ^]^ HIV, cancer, intestinal bowel disease (IBD), infections, and recurrent diarrhea that drastically increase energy requirements and/or interfere with nutrient absorption and metabolism are also known to be leading causes of undernutrition in humans. These coupled with the multiple organ impairments and metabolic dysregulation sporadically reported in undernourished individuals, underscore the complexity of the etiology and mechanism of the disease state.^[^ ^3^, ^7^ ^]^ Over the years, some advances have been made in understanding the mechanism of undernutrition. Recently, it was uncovered that peroxisomal and mitochondrial dysfunction underpins undernutrition‐associated liver steatosis and ATP depletion,^[^ ^8^ ^]^ raising the possibility of targeting the restoration of mitochondrial function in combatting undernutrition. In spite of these, current dietary therapy still relies on the supply of high protein high calorie formulas for nutritional recovery that has achieved relative reduction in mortality but restoration of impaired organs remains elusive resulting in significant relapse of cases.^[^ ^9^ ^]^ This phenomenon could be partly attributed to the lack of appropriate model which is not only closest to humans, phylogenetically, but also capable of recapitulating the clinical state of undernutrition for mechanistic exploration. So far, rodent models do not adequately reflect the pathological complexity in undernourished humans, while a piglet model of undernutrition presented with even elevated albumin levels contrary to the hypoalbuminemia observed in undernourished individuals.^[^ ^10^ ^]^

Non‐human primates (NHPs) are phylogenetically closest to humans, having similar genomes, circadian rhythms, nutrient requirements, and feeding patterns.^[^ ^11^ ^]^ About 60 years ago, baboon was used to mimic partial undernutrition symptoms, and proved that NHPs could serve as a valuable tool to study undernutrition.^[^ ^12^ ^]^ However, systematic research using NHPs, to explore the pathophysiological mechanism of undernutrition and subsequently used as the basis for designing effective therapeutic strategies, is still unavailable.

Using 1636 *Macaca fascicularis* monkeys, herein we developed a weight‐for‐age z‐score (WAZ) criteria which successfully identified undernourished NHPs (U‐NHPs) epitomizing human undernutrition manifestation. Taking advantage of this model, we explored the underlining mechanism using biochemical, pathological, and multi‐omics analytic approaches. Based on the observed severe hepatic metabolic dysregulation and impaired mitochondrial function, we designed a novel soy peptide supplementation strategy capable of reprograming hepatic lipid metabolism and restoring mitochondrial function to attenuate undernutrition.

## 2 Results

### 2.1 Establishment of Undernutrition Criteria in NHPs

Compared to the healthy‐looking types, the non‐thriving monkeys were significantly emaciated with body fat and muscle loss, visible rib bones, loss of hair pigmentation and presented with fatigue, irritability and lethargy (Figure S1A). From the initial assessment, we observed a general decrease in total food intake among the non‐thriving NHPs relative to their normal counterparts (Figure S1B, Supporting Information). We also observed that non‐thriving NHPs had significant low movement as indicated by the distance covered within a 24 h period compared to their normal counterparts, implying a significant reduction in activity level. Undoubtedly, these are akin to signs of undernutrition as observed in humans (Figure S1C–E). The records further showed that these monkeys also experienced on the average 8.5 times recurrent diarrhea episodes for the 30 months preceding the study (Figure S1F). To further understand the possible reasons for the observed emaciation, we performed fecal occult blood testing (FOBT), a non‐invasive diagnostic test for signs of gastrointestinal diseases in humans. The results showed that the non‐thriving monkeys (3.38 ± 1.27) had significant intestinal bleeding compared to the healthy‐looking (0.67 ± 0.44, *P* < 0.001) counterparts (Figure S1G, Supporting Information). Moreover, postmortem examination upon the death of some of the non‐thriving monkeys uncovered significant intestinal inflammation characteristic of inflammatory bowel disease (IBD) in both the colon (8.55 ± 0.54 verses 1.00 ± 0.82, *P* < 0.001) and the ileum (4.12 ± 1.03 verses 0.33 ± 0.47, *P* < 0.001) compared to their healthy‐looking counterparts (Figure S1H‐K). It is worth noting that recurrent diarrhea and IBD are significant risk factors and causes of human undernutrition for their ability to impair nutrient absorption and metabolism.^[^ ^13^ ^]^

**Figure 1:**
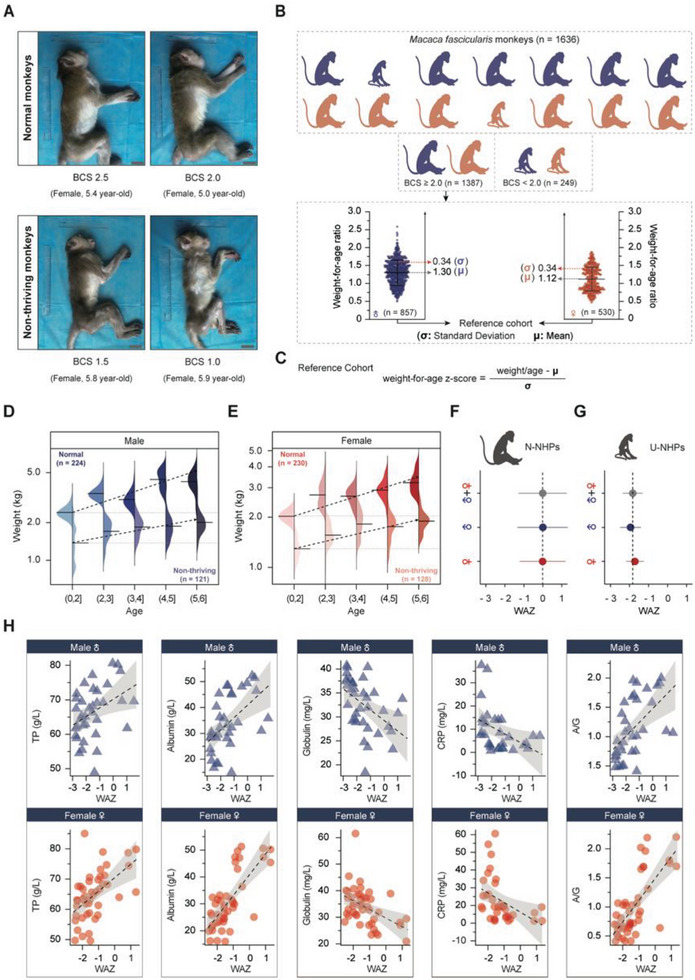
Characterizing human undernutrition manifestation in undernourished NHPs. A) Representative images of NHPs of different BCS scores. Scale bar: 5 cm. B) Top panel: Schematic representation of the selection and grouping of NHP monkeys with BCS ≥ 2.0, (n = 1387) and BCS < 2.0, (n = 249). Bottom panel: Mean and standard deviation of the weight‐for‐age ratio of monkeys with BCS ≥ 2.0 (reference population); Left: Males (n = 857). Right: Females (n = 530). C) Equation for the calculation of weight‐for‐age z‐score (WAZ). D,E) Weight distribution by age of the study cohort for males with BCS ≥ 2.0 (n = 224) and BCS < 2.0 (n = 121) (D), and females with BCS ≥ 2.0 (n = 230) and BCS < 2.0 (n = 128) (E). F) WAZ of NHPs of BCS ≥ 2.0 (0.0, CI: −0.17 to 0.18) consistent with that of healthy humans. G) WAZ of NHPs of BCS < 2.0 showing significant deviation from WAZ (0.0) of healthy humans. H) Linear regression analysis showing the relationship between WAZ score and (from left to right) TP, Albumin, Globulin, CRP, and A/G. (N‐NHP = 20; U‐NHP = 60). Statistical analysis was done using an independent sample *t‐test*. *P < 0.05; **P < 0.01; ***P < 0.001. NHP: Non‐human primate; N‐NHP: Normal non‐human primate; U‐NHP: Undernourished non‐human primate; BCS: Body condition score; WAZ: Weight‐for‐age z‐score; TP: Total protein; CRP: C‐reactive protein; A/G: Albumin/globulin ratio.

For uniformity in identifying the two observed phenotypes, monkeys were classified into the normal and non‐thriving groups based on the body condition scoring (BCS) system.^[^ ^14^ ^]^ BCS is a semi‐quantitative and highly subjective 1 – 5 scoring system, used to assess the lean body mass and body fat of mammalian species. Generally, higher scores (BCS ≥ 2.0) indicate non‐emaciated body status and lower scores (BCS < 2.0) represent emaciated body status.^[^ ^14^ ^]^ Here, from a total of 1636 *Macaca fascicularis* monkeys, we identified 1387 normal (BCS ≥ 2.0) and 249 non‐thriving (BCS < 2.0) monkeys exhibiting the undernutrition phenotype (**Figure** **1**A,B). We then applied the WHO‐recognized weight‐for‐age z‐score (WAZ), an undernutrition diagnosing criteria in humans,^[^ ^15^ ^]^ to measure underweight status in the non‐thriving NHP population. WAZ is defined as the number of standard deviations below or above the reference mean weight. For NHPs, reference mean weight has not been established. Thus, to calculate the mean weight‐for‐age ratio of normal monkeys, a total of 1387 monkeys comprising 857 males and 530 females was selected as the reference cohort (the reference population mean, µ, Figure 1B; Figure S1L; Table S1). The weight‐for‐age ratio of the reference cohort was calculated to be 1.30 ± 0.34 and 1.12 ± 0.34 for males and females, respectively (Figure 1B; Table S1, Supporting Information).

To calculate WAZ scores of NHPs using the obtained reference cohort, we selected the non‐thriving monkeys (121 males and 128 females, BCS < 2.0), and approximately double the number of age‐ and sex‐matched normal monkeys (224 males and 230 females, BCS ≥ 2.0) (Figure 1C–E and Table S2,S3). The WAZ of the normal monkeys was 0.00 (CI: −0.17 to 0.18) (Figure 1F and Table S4), which is consistent with the WHO‐developed international growth standards.^[^ ^2^, ^15^ ^]^ The non‐thriving monkeys had a significantly low mean WAZ of −1.83 (CI: −1.89 to −1.76, *P* < 0.001), affirming undernutrition status (Figure 1G and Table S4). Thus, for the first time, we determined WAZ ≤ −1.83 as the criteria for identifying undernutrition in NHPs.

WHO also recommends mid‐upper‐arm circumference (MUAC), an independent predictor of growth failure, for identifying undernutrition.^[^ ^15^ ^]^ Here, we found that MUAC for non‐thriving male NHPs (mean = 8.1 cm; CI: 7.7 – 8.9) was lower than their normal counterparts (mean = 9.4 cm; CI: 9.3 – 9.6, *P* < 0.001) (Figure S2A and Table S5,S6). Also, non‐thriving female NHPs had lower MUAC (mean = 8.1 cm, CI: 7.8 – 8.3) compared to the normal ones (mean = 9.5 cm, CI: 9.2 – 9.8, *P* < 0.001) (Figure S2C and Table S5,S6, Supporting Information). Besides, other anthropometric measurements including head circumference, subscapular and triceps skinfolds further confirmed U‐NHPs presented with substantial undernourishment (Figure S2B‐D and Table S5,S6).

Taken together, the WAZ (≤ −1.83) criteria and MUAC values obtained based on 1636 monkeys could be applied to identify undernourished *Macaca fascicularis* NHPs (U‐NHPs) as a model of human undernutrition.

### 2.2 U‐NHPs Showed Abnormal Serum Biochemistry Profile

Serum biochemical parameters have been used in conjunction with thorough physical examination in diagnosing undernutrition.^[^ ^16^ ^]^ In this study, we found remarkable decrease in albumin level (47.13 ± 4.68 versus 27.16 ± 6.52 g L⁻^1^, *P* < 0.0001) and total protein level (72.95 ± 7.31 versus 62.98 ± 6.55 g L⁻^1^, *P* < 0.0001) (Figure S2E,G, Supporting Information) in U‐NHPs. This is in agreement with the hypoalbuminemia observed in undernourished humans,^[^ ^17^ ^]^ proving that U‐NHPs, compared with undernourished piglets with hyperalbuminemia,^[^ ^10^ ^]^ could better mimic the human condition. In addition, glucose level was not significantly impacted by the undernutrition status (Figure S2H, Supporting Information), similar to what is found in undernourished individual.^[^ ^18^ ^]^ Serum triglyceride (TG) was found increased in U‐NHPs, while total cholesterol and low‐density lipoprotein cholesterol (LDLC) decreased, and density lipoprotein cholesterol (HDLC) remained relatively stable (Figure S2I‐L). Moreover, aspartate transferase (AST) was significantly elevated, while alanine aminotransferase (ALT) remained relatively unchanged in U‐NHPs compared to N‐NHPs (Figure S2M,N, Supporting Information), hinting the occurrence of liver irritations. In addition, blood urea nitrogen (BUN) and creatinine levels both decreased in U‐NHPs (Figure S2O,P). Furthermore, the inflammatory indices globulin and C‐reactive protein (CRP) were also higher whiles albumin/globulin ratio was lower in U‐NHPs compared to their normal counterparts (Figure S2Q–S, Supporting Information). These findings support the clinical assertion that low levels of albumin and total protein could reflect not only suboptimal nutritional status, but also suggest possible systemic inflammation.^[^ ^16^ ^]^ Predictably, linear regression analysis of biochemical parameters and WAZ demonstrated that serum albumin and total protein levels correlated positively with WAZ, while globulin and CRP levels correlated negatively with WAZ (Figure 1H; Table S7–S10, Supporting Information), indicating that systemic inflammatory levels are associated with the severity of undernutrition.

### 2.3 Pathological Analysis Showed Histological Changes in U‐NHP Organs

**Figure 2:**
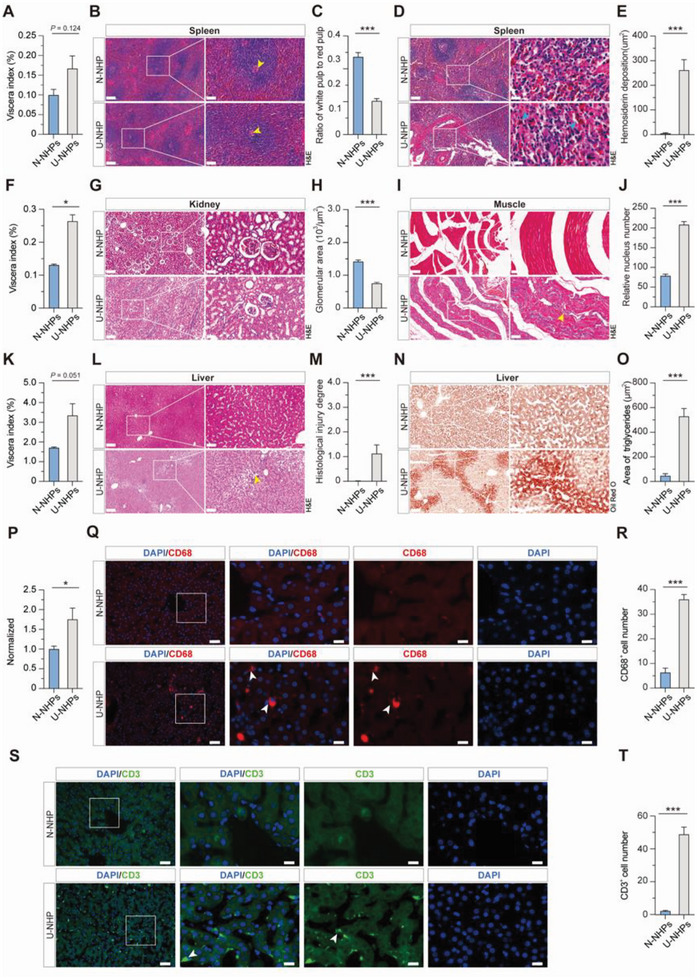
Histological changes in U‐NHP organs. A) Viscera index of spleen between N‐NHPs and U‐NHPs. B) H&E staining of spleen sections showing white pulp atrophy along with expansion of red pulp. Scale bar: 200 µm (left panel) and 50 µm (right panel). C) Quantification analysis of white pulp‐to‐red pulp ratio in the spleen. D) H&E staining of spleen sections showing hemosiderin deposition in U‐NHPs. Scale bar: 100 µm (left panel) and 20 µm (right panel). E) Quantification of relative hemosiderin deposition between N‐NHPs and U‐NHPs. F) Viscera index of kidney between N‐NHPs and U‐NHPs. G) H&E staining of kidney sections showing glomerular atrophy. Scale bar: 200 µm (left panel) and 50 µm (right panel). H) Relative glomerular area in the kidney of N‐NHPs and U‐NHPs. I) H&E staining of muscle sections showing an increased number of nuclei. Scale bar: 200 µm (left panel) and 50 µm (right panel). J) Relative number of nuclei in the muscle between N‐NHPs and U‐NHPs. K) Viscera index of liver between N‐NHPs and U‐NHPs. L) H&E staining of liver sections showing increased lipid accumulation. Yellow arrow shows neutrophil infiltration. M) Relative histological injury degree score of liver between N‐NHPs and U‐NHPs. N) Oil Red O staining of liver sections. Scale bar: 200 µm (left panel) and 50 µm (right panel). O) Quantification of triglycerides accumulation. P) Triglyceride level normalized to liver weight. Data were pooled from six experiments with triglyceride levels normalized to the N‐NHP group of each experiment. Q) Immunofluorescence staining of CD68^+^ showing activated macrophages. R) Relative CD68^+^ count in liver between the N‐NHPs and U‐NHPs. Scale bar: 500 µm (top panel) and 100 µm (bottom panel). S) Immunofluorescence staining of CD3^+^ showing T cell infiltration in U‐NHP liver. T) Relative CD3^+^ number in liver between the N‐NHPs and U‐NHPs. Scale bar: 500 µm (top panel) and 100 µm (bottom panel). (N‐NHP = 6; U‐NHP = 6). Statistical analysis was done using independent sample *t‐test*. *P < 0.05; **P < 0.01; ***P < 0.001. N‐NHP: Normal non‐human primates; U‐NHP: Undernourished non‐human primates.

The limited availability of pathological dataset on undernutrition‐related organs prompted us to perform Hematoxylin‐Eosin (H&E) staining of frequently reported organs in undernutrition (spleen, kidney, muscle, and liver). In U‐NHPs, atrophy of the splenic white pulp, and corresponding expansion of the red pulp, as well as hyaline degeneration and hemosiderin deposition, were observed in U‐NHPs (**Figure** **2**A‐E), indicating the impaired structural integrity of the spleen. Moreover, splenic weight in U‐NHPs (2.7 ± 1.0 g) was significantly reduced (*P* < 0.05) compared to N‐NHPs (5.1 ± 1.9 g) (Table S11, Supporting Information) in agreement with post‐mortem data from malnourished humans.^[^ ^19^ ^]^ In the kidneys, U‐NHPs presented with glomerulosclerosis, tubular atrophy, and epithelial vacuolization, along with a significant reduction in kidney weight and glomerular area (Figure 2F‐H and Table S11), suggesting that undernutrition could have highly detrimental effects on renal function. Although decreased glomerular filtration rate and general impaired renal function have been reported in undernourished individuals, there is a lack of pathological evidence due to challenges with acquiring biopsy samples. In the muscles, increased muscular atrophy and degeneration of muscle fibers were revealed in U‐NHPs, reflecting severe muscle mass depletion in undernourished humans. While N‐NHPs were characterized by uniform peripheral nuclei, the nuclei in the muscle of U‐NHPs were irregularly shaped and tended to be more centrally distributed in the cytoplasm (Figure 2I,J). Strikingly, U‐NHP muscles were further characterized by fiber necrosis, along with significant lymphocyte and fatty infiltration, indicating the presence of inflammation, which is also observed in undernourished humans.^[^ ^20^ ^]^ Furthermore, liver (54.3 ± 20.6 g) in U‐NHPs tended to weigh less (*P* < 0.05) compared to N‐NHPs (93.0 ± 6.0 g) (Figure 2K and Table S11) although stained liver tissues revealed severe hepatocyte ballooning, vacuolization, and macro vesicular lipid droplets, indicating the occurrence of hepatic steatosis (Figure 2L). In contrast to N‐NHPs, significant neutrophil and lymphocyte infiltration could also be observed in the liver of U‐NHPs. Quantification analysis showed high histological injury degree scores in U‐NHPs relative to their normal counterparts (*P* < 0.001) (Figure 2M). Oil Red O staining and quantification further verified the substantial accumulation of triglycerides in hepatocytes of U‐NHPs (Figure 2N‐P). These confirm the development of hepatic steatosis, a common trait of edematous undernutrition, in U‐NHPs.

Hepatic steatosis is usually accompanied by inflammation,^[^ ^21^ ^]^ thus we performed immunofluorescence (IF) staining of CD68^+^, and found the increased number of CD68^+^ cells in U‐NHP livers (Figure 2Q,R). When the liver is damaged, activated CD68^+^ macrophages can produce various proinflammatory cytokines and chemokines, leading to lymphocytes infiltration.^[^ ^22^ ^]^ CD3^+^, the typical lymphocytes marker was next stained and as expected, we found significant T cell infiltration in the liver of U‐NHPs (Figure 2S,T). It is worth noting that macrophage activation and lymphocytes recruitment have not been previously reported in existing undernutrition animal models.

### 2.4 Transcriptomic Profiling Revealed Dysregulated Biological Response Pathways in U‐NHPs

Molecular level alterations of the significantly impaired organs were further uncovered by transcriptome profiling and biological pathway enrichment analysis. Considering the variability in gene expression among individuals, we utilized a linear mixed model to analyze the impact of tissue variance and individual variance on the expression of each gene. Our findings indicated that individual variance had a limited effect, while tissue variance emerged as the primary factor influencing the pattern of gene expression (Figure S3A, Supporting Information). To further understand the changes in molecular pathways associated with undernutrition, we performed differentially expressed genes (DEGs) analysis of the frequently reported organs in undernutrition (Figure S3B).

In the spleen, the regulation of immune response pathways, such as TNF signaling and NF‐κB signaling pathways, is key for maintaining spleen function.^[^ ^23^ ^]^ In U‐NHPs, we found that upregulated differentially expressed genes (DEGs) were strongly associated with the upregulation of inflammatory process pathways, such as TNF and NF‐κB (Figure S3C). Moreover, upregulation of IL‐17 signaling pathway was also found, further hinting the breakdown of immune tolerance, and thus the promotion of pro‐inflammatory response (Figure S3C, Supporting Information).

The RNA‐binding protein (RBPs) plays an important role in kidney disease including glomerulosclerosis and tubulointerstitial fibrosis. RBPs also control RNA splicing and participate in the formation of ribonucleoprotein complexes that build up spliceosome complex.^[^ ^24^ ^]^ In U‐NHPs, RNA splicing and spliceosomal complex were both upregulated in comparison with N‐NHPs (Figure S3D, Supporting Information), implying that their abnormal regulation could be responsible for the observed glomerulosclerosis and tubular atrophy in undernourished conditions.

Muscle loss in undernutrition is believed to be associated with significant systemic inflammation. Several studies have proposed that the dysregulation of cytokines especially TNF‐ɑ stimulate the degradation of muscle mass. Herein, biological processes relating to inflammation such as TNF production and myeloid leukocyte activation were all upregulated in U‐NHPs (Figure S3E, Supporting Information). Moreover, pathways associated with T cell receptor signaling and chemokine signaling were also upregulated. This is consistent with the observed lymphocyte infiltration, muscular atrophy, and degeneration of muscle fibers observed in U‐NHP muscle.

**Figure 3:**
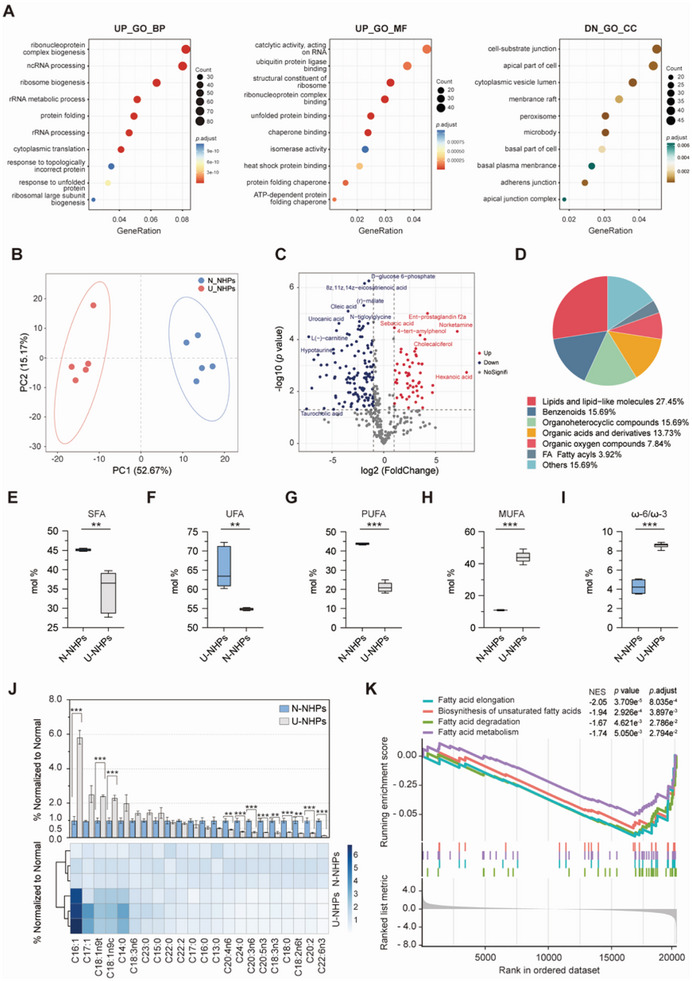
U‐NHPs are characterized by dysregulation of fatty acid metabolism. A) Gene ontology (GO) analysis of metabolic pathways associated with differentially expressed genes in the liver (n = 3 for each group). B) Principal component analysis (PCA) showing delineation of hepatic metabolite abundance between N‐NHPs and U‐NHPs (n = 5 for each group). C) Volcanic plots of differential abundance of liver metabolites between N‐NHPs and U‐NHPS. Red color shows metabolites with significantly increased abundance while blue color denotes metabolites with significantly decreased abundance in U‐NHPs in comparison with N‐NHPs. D) The classification of identified hepatic metabolites showing that lipid and lipid molecules account for the largest differentially abundant metabolites in the liver. E‐I) Relative abundance of SFA (E), UFA (F), PUFA, (G) and MUFA (H) in the liver of N‐NHPs and U‐NHPs (n = 6 for each group) determined by gas chromatography‐mass spectrometry (GC‐MS/MS). I) Ratio of hepatic ω−6/ω−3 between N‐NHPs and U‐NHPs. J) The relative abundances of MUFA and PUFA subtypes (n = 6 for each group). K) GSEA plot of differentially enriched pathways associated with fatty acid metabolism between N‐NHPs and U‐NHPs (n = 6 for each group). Statistical significance was determined using ANOVA with post hoc Dunnett's test and indicated by as *P < 0.05; **P < 0.01; ***P < 0.001. UP: Up regulated; DN: Down regulated; GO: Gene ontology; BP: Biological process; MF: Molecular function; CC: Cellular component. N‐NHPs: Normal non‐human primates; U‐NHPs: Undernourished non‐human primates; SFA: Saturated fatty acids; UFA: Unsaturated fatty acids; PUFA: Polyunsaturated fatty acids; MUFA: Monounsaturated fatty acid; GSEA: Gene set enrichment analysis.

With regards to liver, pathways responsible for cell polarity including apical part of cell, membrane raft and basal part of cell were all downregulated (**Figure** **3**A), consistent with the observed hepatocyte ballooning in U‐NHP liver by H&E staining (Figure 2I). In addition, the upregulation of ubiquitin protein ligase binding, ribonucleoprotein complex binding, heat shock protein binding and protein folding chaperone were revealed in U‐NHPs (Figure 3A), indicating the activation of unfolding protein response (UPR). The upregulated UPR process under liver mitochondrial stress in U‐NHPs could have contributed to the development of hepatic lipid accumulation.

### 2.5 Hepatic Steatosis in U‐NHPs Reflects Dysregulated Fatty Acid Metabolism

Given the multiple metabolic process negatively regulated in the liver as revealed by the transcriptomic analysis, and the central role of the liver in metabolism, we envisaged that such an acute deterioration of hepatic function in undernutrition may be reflected in abnormal metabolic profiles. With the liver as the hub of nutrient metabolism, uncovering such atypical hepatic metabolites could be significant for undernutrition clinical practice by providing further insight about the disease pathology and also for monitoring prognosis. Thus, we performed metabolomics analysis to understand metabolic changes within the liver. The principal component analysis (PCA) showed a clear delineation of the hepatic metabolites between N‐NHPs and U‐NHPs, implying a significant difference in accumulated metabolites (Figure 3B) lending credence to substantial deviation in hepatic function in undernutrition. Indeed, a volcanic plot constructed using mass features with normalized abundance greater 1 to minimize false positives confirmed that N‐NHPs accumulated a set of metabolites considerably different from that of U‐NHPs (Figure 3C, Figure S4A). The predominant metabolites (normalized abundance > 5) with decreased abundance in U‐NHPs included L(‐)‐carnitine, hypotaurine, pregnenolone and N‐tiglylglycine whiles cholecalciferol and norketamine increased in abundance (Figure 3C, Figure S4A).

In all, a total of 376 metabolites were identified by the untargeted metabolomics analysis with lipid and lipid‐like molecules constituting the largest group (27.45%) of differentially accumulated metabolites between the N‐NHPs and U‐NHPs (Figure 3D).

Lipid accumulation is associated with changes in the relative composition of fatty acid (FA) moieties, which contribute to the pathogenesis of hepatic steatosis.^[^ ^25^ ^]^ We thus, profiled fatty acid spectrum using gas chromatography‐mass spectrometry (GC‐MS/MS), and found significant decrease in percent saturated fatty acids (SFAs) and a concurrent increase in percent unsaturated fatty acids (UFAs) in U‐NHPs (Figure 3E,F). Although polyunsaturated fatty acids (PUFAs) decreased, there was a higher compensatory increase in monounsaturated fatty acids (MUFAs) (Figure 3G,H), which culminated in the overall increase in UFAs. Elevated ω−6/ω−3 ratio has been used as an inflammatory marker in diseases associated with hepatic steatosis such as NAFLD and NASH.^[^ ^26^ ^]^ Herein, we found significant elevation of ω−6/ω−3 ratio in U‐NHPs compared to N‐NHPs (Figure 3I), further emphasizing undernutrition associated inflammation. To ascertain which specific MUFA and PUFA moieties are culpable in undernutrition‐associated hepatic steatosis, the abundance of individual FAs was further examined (Figure 3J). The results showed that MUFAs, including Palmitoleic acid (C16:1), trans‐9‐Octadecanoic acid (C18:1n9t) and cis‐9‐Octadecanoic acid (C18:1n9c) were all strikingly elevated in U‐NHPs compared with their healthy counterparts. In contrast, PUFAs including cis‐11,14‐Eicosadienoic acid (C20:2), cis‐8,11,14‐Eicosatrienoic acid (C20:3n6), 5,8,11,14‐Eicosatetraenoic acid (C20:4n6) and 9,12,15‐Octadecatrienoic acid (C18:3n3) except 6,9,12‐Octadecatrienoic acid (C18:3n6) were significantly decreased in U‐NHPs. In line with this, we found that pathways associated with fatty acid metabolism were downregulated in the U‐NHPs (Figure 3K; Table S12, Supporting Information), which was in agreement with previous study that malnutrition could alter lipid metabolism and trigger hepatic steatosis.^[^ ^25^ ^]^ Accordingly, we observed a significant correlation between undernutrition severity, serum metabolites, and liver metabolites. We found significant negative association between levulinic acid, cholecalciferol, tyrosaylalanine, and hexanoic acid, and both WAZ scores and serum albumin levels while D‐a‐tocopherol, hypotaurine and epinephrine were positively associated with both WAZ scores and serum albumin levels (Figure S4B, Supporting Information).

### 2.6 Mitochondrial Dysfunction Drives Hepatic Steatosis in U‐NHPs

**Figure 4:**
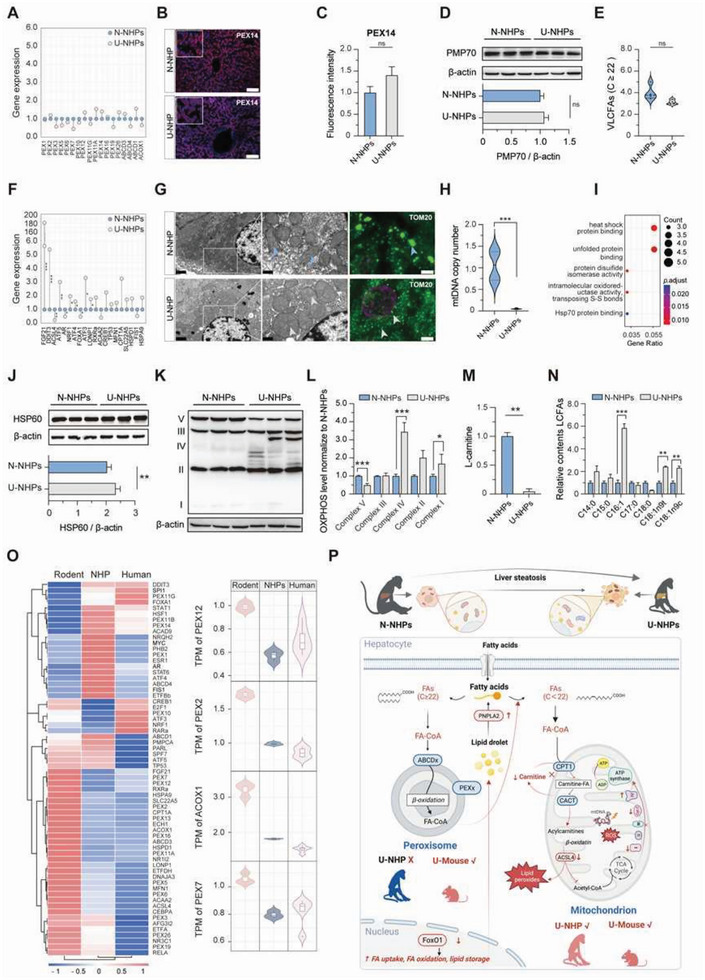
Multi‐dimensional analysis of liver tissues revealed mitochondrial dysfunction underlines lipid dysregulation in U‐NHPs. A) Expression of genes associated with peroxisomal activity in the liver of N‐NHPs and U‐NHPs. B) Immunofluorescent staining of peroxisome marker PEX14 in liver. Scale bar: 100 µm. C) Relative fluorescence intensity of peroxisome marker, PEX14. D) Western Blot analysis and quantification of protein levels of PMP70. E) Differences in hepatic very long‐chain fatty acid (carbon chain length ≥ 22) between N‐NHPs and U‐NHPs. F) Expression of genes associated with mitochondrial activity in the liver of N‐NHPs and U‐NHPs. G) TEM of liver periportal hepatocytes showing mitochondrial morphology (Scale bar: 1 µm, left panel; 0.5 µm, right panel) and immunofluorescent staining of TOM20 (scale bar: 100 µm). of liver. H) Relative levels of hepatic mtDNA copy number. I) Metabolic pathways associated with upregulated genes in the liver. J) Western Blot analysis and quantification showing protein levels of the mitochondrial chaperone HSP60 (Bottom panel). K) Western Blot analysis of OXPHOS complexes I‐V. L) Quantification of the relative protein levels of OXPHOS complexes I‐V between N‐NHPs and U‐NHPs. M) Relative levels of hepatic L‐carnitine. N) Relative levels of LCFAs between N‐NHP and U‐NHPs. O) Specie differences in the expression pattern of peroxisomal and mitochondrial‐associated genes under normal physiological conditions among rodents, NHPs and humans. P) Schematic diagram of the mechanism involved in undernutrition‐associated lipid metabolic dysregulation and hepatic steatosis in U‐NHP livers. (N‐NHP = 6; U‐NHP = 6). Statistical analysis was done using independent sample *t‐test*. *P < 0.05; **P < 0.01; ***P < 0.001. N‐NHP: Normal non‐human primates; U‐NHPs: Undernourished non‐human primates; VLCFAs: Very long‐chain fatty acids; LCFAs: Long‐chain fatty acids.

Hepatic steatosis is often attributed to decreased hepatic fatty acid oxidation (FAO),^[^ ^27^ ^]^ mainly resulting from impairments in hepatic peroxisomal and/or mitochondrial function. To investigate the veracity of this in undernutrition conditions, we first examined the expression level of genes associated peroxisomal biogenesis and function, and the results showed that PEX2, PEX7, PEX12, PEX14, and ACOx1 etc. remained unchanged in undernutrition state as revealed by U‐NHPs transcriptomic profiling (**Figure** **4**A). IF staining of PEX14, the peroxisomal biogenesis marker, further confirmed no obvious alteration (*P* > 0.05) of peroxisome level in U‐NHPs (Figure 4B,C), implying the limited participation of peroxisome in undernutrition‐related hepatic steatosis. This assertion was further affirmed by the peroxisome membrane marker (PMP70) expression analysis showing no apparent difference in peroxisomal morphology (Figure 4D). Since peroxisome is responsible for the β‐oxidation of very long‐chain fatty acids (VLCFA, carbon chain length ≥ 22), the unchanged VLCFA level further proved that peroxisomal activity was not affected under malnourished conditions in U‐NHPs (Figure 4E).

Subsequently, we evaluated the mitochondrial function‐associated genes to uncover if mitochondrial dysfunction was involved in hepatic steatosis in U‐NHPs. Intriguingly, genes associated with mitochondrial fatty acid oxidation including DNA damage inducible transcript 3 (DDIT3), lon peptidase 1 (LONP1) and activating transcription factor 3 (ATF3) among others, were upregulated in U‐NHPs (Figure 4F). Significant accumulation of deformed mitochondria in the liver of U‐NHPs were noticed by transmission electron microscopy (TEM) imaging (Figure 4G). Also, the reduction of immunofluorensence intensity of mitochondria outer membrane protein (TOM20), (Figure 4G) suggested an obvious reduction in mitochondrial mass and further confirmed abnormal mitochondria morphology. Moreover, significant decrease in hepatic mitochondrial DNA (mtDNA) copy number in U‐NHPs signified substantial mitochondrial loss (Figure 4H). Taken together, the above results clearly emphasized that mitochondria were involved in the observed hepatic steatosis in U‐NHPs.

In general, hepatic lipid accumulation is associated with aberrant fatty acid oxidation, resulting from abnormal mitochondria response under stress. In U‐NHPs, heat shock protein binding and unfolded protein binding pathways were upregulated (Figure 4I; Figure S5A, Supporting Information), consistent with the observed increase in mitochondrial unfolded protein response (UPR^mt^) transcription factors (DDIT3, ATF4, and ATF5).^[^ ^28^, ^29^, ^30^ ^]^ The occurrence of UPR^mt^ was further confirmed by the elevated protein levels of heat shock protein 60 (HSP60), a mitochondrial chaperone, in U‐NHP livers (Figure 4J). Generally, oxidative phosphorylation (OXPHOS) perturbation is considered to cause mitochondrial stress,^[^ ^31^ ^]^ thereby activating UPR^mt^. Predictably, Western Blot (WB) analysis of the protein level of OXPHOS complexes showed that complex V decreased while complexes IV and II increased in U‐NHPs (Figure 4K,L). On the contrary, disruption in complex I activity has often been reported in prior undernutrition rodent models.^[^ ^8^, ^32^ ^]^ Intriguingly, the expression of genes associated with complex I (NDUFAF5, NDUFA12, NDUFB1) did not vary between U‐NHPs and N‐NHPs but the complex IV‐related gene, NDUFA4L2, significantly decreased in U‐NHPs (Figure S5B–E, Supporting Information), affirming our observation that complex I may be less involved in mitochondrial dysfunction in undernutrition monkeys. More importantly, the OXPHOS complex in U‐NHPs showed multiple undefined specific bands which were nonexistent in N‐NHPs (Figure 4K), suggesting increased hydrolysis of OXPHOS complex in undernutrition. In addition, the levels of LC3‐II protein in the liver of U‐NHPs are significantly increased, indicating an activation of autophagy in response to undernutrition. The reduction in p62 levels further confirms this activation, suggesting an enhanced autophagic flux in the liver of U‐NHPs (Figure S5F–H, Supporting Information). Furthermore, we observed a sharp decrease in the abundance of L‐carnitine levels, shuttle molecule which facilitates the entry of long‐chain fatty acids (LCFAs, 12 < carbon chain length < 22) into the mitochondrial for oxidation, in U‐NHPs (Figure 4M). Consistent with this, a deterioration in LCFAs oxidation (Figure 4N) resulted in increased lipid accumulation, indicating hepatic mitochondrial dysfunction in U‐NHPs.

The current evidence showing the unlikely involvement of the peroxisome in steatosis in U‐NHPs is contrary to earlier report in a rodent model,^[^ ^8^ ^]^ which might be due to specific differences in expression pattern of lipid metabolism genes. We thus compared the expression level of mitochondrial and peroxisome genes associated with hepatic lipid metabolism between rodents, NHPs, and humans. Interestingly, the results showed that under physiological conditions, the expression pattern of most genes (PEX2, PEX7, PEX12, PEX14, ACOx1 etc.) regulating peroxisomal β‐oxidation were similar between NHPs and humans, and very distinct for rodents (Figure 4O). The differential pathway regulation under stress condition‐associated lipid metabolism between rodents, NHPs and humans, were then analyzed. The overall results showed that the global alterations in metabolic pathways in NHPs were even more akin to those of humans and substantially differing from those in rodent models (Figure S5I). It was further revealed that alterations in pathways associated fatty liver in both humans and NHPs (e.g., oxidoreductase activity, signal receptor binding, and regulation of heme production) are different from those in rodents (e.g., transport activity and hydrolase activity) (Figure S6A‐C). Thus, NHPs could more closely recapitulate lipid metabolic disorder phenotypes in humans compared to rodent models.

In brief, the evidence demonstrate that undernutrition associated hepatic steatosis is primarily due to mitochondrial dysfunction with limited participation of hepatic peroxisome contrary to what is observed in rodent models. Mitochondrial dysfunction is principally characterized by OXPHOS perturbation and mitochondrial absence in U‐NHPs, given rise to decreased fatty acid oxidation which then leads to hepatic steatosis in U‐NHPs (Figure 4P).

### 2.7 Soy Peptide Supplementation Reverses Undernutrition Symptom in U‐NHPs

**Figure 5:**
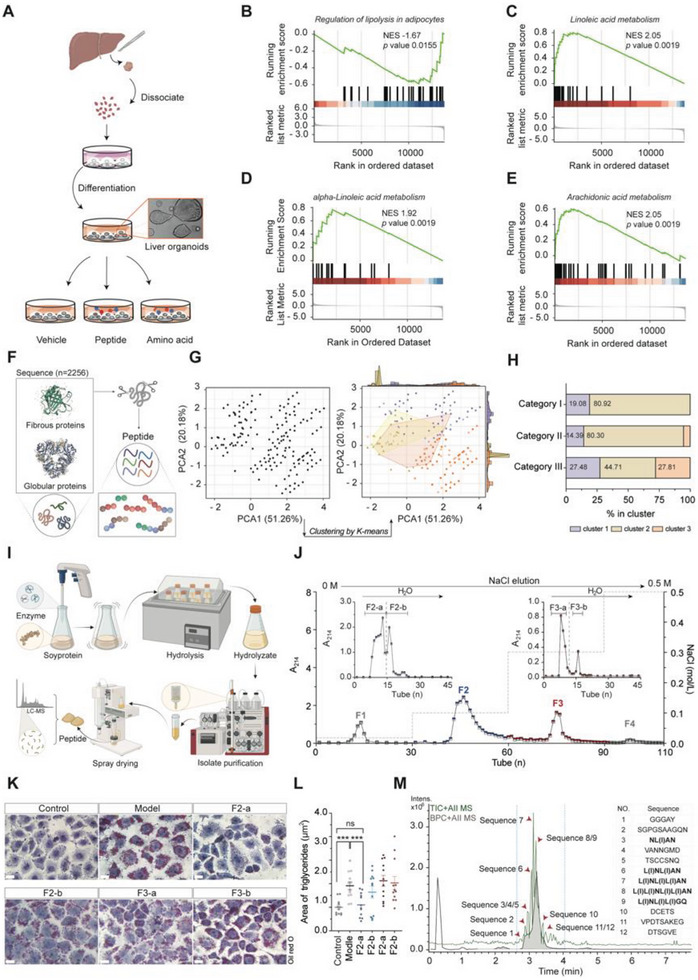
Hypolipidemic potential of bioactive soy peptides. A) Schematic diagram for the experiment on the hepatic lipid regulation function of peptides in liver organoid. B–E), pairwise comparison of GSEA plot (score curves) showing significant enrichment in pathways associated with regulation of lipolysis in adipocytes between peptide and vehicle control (B), Linoleic acid metabolism (C), Alpha‐Linolenic acid metabolism (D) and Arachidonic acid metabolism (E) between peptide and amino acid treatments. The green curve corresponds to the enrichment scores curve, which is the running sum of the weighted enrichment score obtained from the GSEA software, while the normalized enrichment score (NES) and the corresponding p‐values testing the significance of each comparison are shown. F) Schematic representation for the acquisition of protein sequences and in silico prediction of simulated digestion‐derived peptides. G,H) Clustering of simulated digestion‐derived peptides from different protein sources. I) Workflow for the preparation of soy peptide. J) Separation and purification of soybean peptides using DEAE‐52 ion exchange resin column. K) Oil Red O staining of LO2 steatosis model showing hepatic lipid‐accumulation relieving potential of soypeptide fractions. L) Relative TG content in LO2 steatosis model upon treatment with purified soypeptide fractions. M) Chromatogram and peak identification of the MS/MS spectrum and the amino acid sequence of the most abundant peptide of the Sephadex G‐15 purified fraction. Scale bar: 10 µm. Statistical analysis was done using independent sample *t‐test*. ***P < 0.001. GSEA: Gene Set Enrichment Analysis. TG: Triglyceride.

Based on the atypical hepatic lipid metabolism in U‐NHPs, and our prior extensive work on bioactive peptides,^[^ ^33^, ^34^ ^]^ we reasoned the potential use of peptides for undernutrition intervention, by targeting hepatic lipid metabolism. To further test the hepatic hypolipidemic bioactivity of peptides, human liver‐organoids were incubated with peptides, and transcriptome analysis showed that lipid metabolic pathways (e.g., linoleic acid, alpha‐linolenic acid, and arachidonic acid metabolism, lipolysis in adipocytes, etc.) were remarkably altered (**Figure** **5**A‐E), affirming that peptides could regulate hepatic lipid metabolism.

In general, peptides encrypted within their parent protein sequences could be released upon gastrointestinal (GI) digestion. Significantly, different protein sources may yield peptides of varying structural, chemical, and functional orientations.^[^ ^35^ ^]^ As per WHO recommendation, current undernutrition therapeutic diets usually contain dairy and legumes (plant)‐proteins.^[^ ^36^ ^]^ Here, the Fasta sequences of 2256 proteins from Uniprot protein library were clusterd by GibbsCluster algorithm to acquire available peptide sequences (Figure 5F; Table S13, Supporting Information). It was found that peptide sequences released from dairy and plant proteins (soybean, pea, peanut, oat etc.) were similar (>80% similarity), hinting potential comparable functions (Figure 5G,H). In particular, soybean recommended by WHO as based protein‐source for treating undernutrition^[^ ^36^ ^]^ was proven to be a good resource for generating biopeptides for attenuating lipid dysregulation.^[^ ^37^ ^]^ Herein, controlled limited proteolysis technique was applied to release the bioactive soy peptide sequences encrypted in the parent soy protein chain (Figure 5I). The capacity of the obtained soy peptides to modulate hepatic lipid metabolism was further confirmed using in vitro LO2 steatosis model. Then, the soy peptides were subjected to extensive purification to further enhance its bioactivity, and the main fraction found to be most effective in attenuating triglyceride accumulation and reducing lipid droplet area in hepatocytes (Figure 5J–L) was also found to be highly enriched in branched chain amino acids (Figure 5 M; Figure S7A, Supporting Information).

The structural characteristics of our soy peptides were further analyzed by mapping the chemical molecular descriptors, and unsurprisingly (Table S14), the PCA analysis revealed that they shared similar chemical characteristics with previously reported hypolipidemic peptides (Figure S7B‐D). Notably, the soy peptides showed enrichment in NHOH and NO groups, along with H^+^ acceptors and donors, relating to the ease of electron transfer for establishing redox balance. It is widely acknowledged that redox imbalance strongly affects mitochondrial function and fatty acid oxidation,^[^ ^38^ ^]^ given further credence that our soy peptides are strong candidates for modulating lipid metabolism.

**Figure 6:**
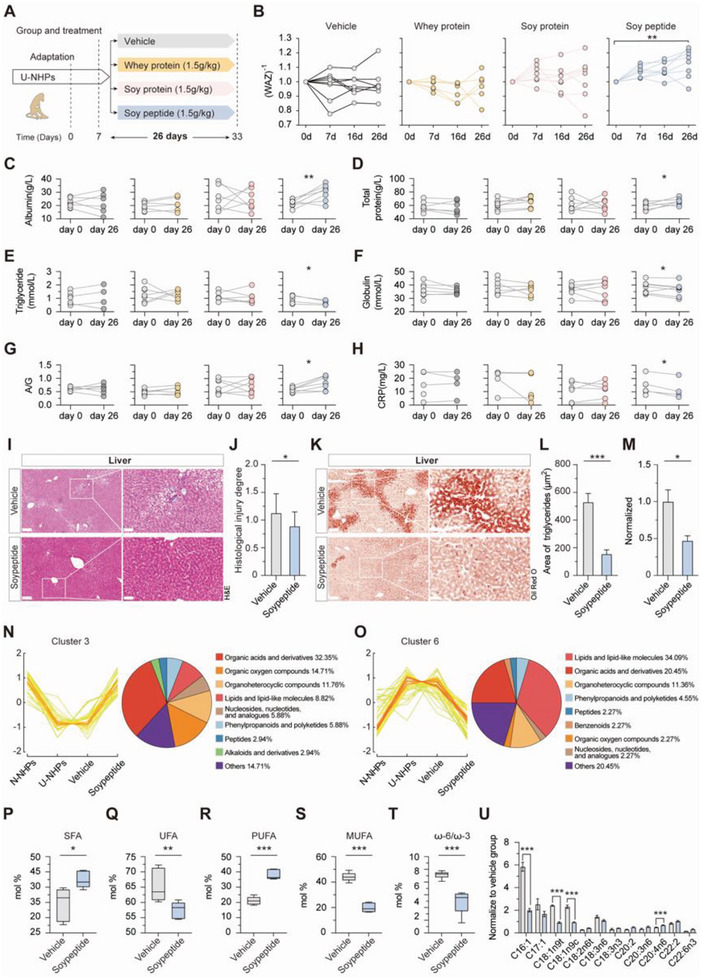
Soy peptide supplementation alleviates hepatic steatosis and mitigates undernutrition in U‐NHPs. A) Schematic of the experimental design for the treatment in U‐NHPs (n = 32). Each group namely vehicle (average age = 4.3 years and WAZ = −2.23), whey protein (average age = 3.9 years and WAZ = −2.20), soy protein (average age = 4.4 years and WAZ = −2.35), and soy peptide (average age = 4.3 years and WAZ = −2.49) consisted of 8 U‐NHPs, with no significant difference in gender. B) Changes in WAZ scores in the vehicle‐treated U‐NHPs (F value = 0.0013; *P* = 0.9718), the soy protein‐treated U‐NHPs (F value = 0.0052; *P* = 0.9428), and the soy peptide‐treated U‐NHPs (F value = 18.7121; *P* = 2e‐04). Values were normalized to baseline (day 0) and inverse values plotted. Analysis was done using ANOVA. (n = 8 for each group). C–H), Changes in levels of Albumin (C), TP (D), TG (E), Globulin (F), A/G (G) and CRP (H) from left to right: vehicle‐treated, whey protein‐treated, soy protein‐treated and soy peptide‐treated U‐NHPs (n = 4 – 8). I), H&E staining of liver sections showing reduced intracellular vacuolation in the soy peptide treated group. n = 6 for each group. Scale bar: 200 µm (left panel) and 50 µm (right panel). J) Quantification of histological injury degree. K) Oil Red O staining of liver sections showing levels of TG accumulation. Scale bar: 200 µm (left panel) and 50 µm (right panel). L) Quantification of relative area of TG accumulation. M) Triglyceride level normalized to liver weight. Data were pooled from six experiments with triglyceride levels normalized to the U‐NHP group of each experiment. N) Fuzzy c‐means clustering showing similar cluster of increased abundance of liver metabolites between N‐NHPs and soy peptide‐treated U‐NHPs distinct from U‐NHPs. O) Fuzzy c‐means clustering showing similar cluster of decreased abundance of liver metabolites between N‐NHPs and soy peptide‐treated U‐NHPs distinct from U‐NHPs. P–S) Relative abundance of SFA (P), UFA (Q), PUFA (R), MUFA (S), and ω−6/ω−3 ratio (T) in the liver between vehicle and soypeptide. U) Fatty acid moieties levels upon treatment with soy peptide. Within group statistical analysis was done using paired sample *t‐test*. *P < 0.05; (n = 6 for each group). U‐NHPs: Undernourished non‐human primates; WAZ: Weight‐for‐age z‐score. TP: Total protein; TG: Triglycerides; TC: Total cholesterol; ALT: Alanine aminotransferase; AST: Aspartate transferase; HDLC: High density lipoprotein cholesterol; LDLC: Low density lipoprotein cholesterol; BUN: Blood urea nitrogen; A/G: Albumin/globulin ratio; CRP: C‐reactive protein. Normal: N‐NHPs; vehicle: U‐NHPs; soypeptide: U‐NHPs‐treated with soypeptides.

Thus, U‐NHPs were supplemented with soypeptide (1.5 g kg⁻^1^/day) using equal amounts of soy protein as control with no differences in nitrogen content and amino acid composition and further compared with equal amount of whey protein, which is recommended by WHO for the treatment of undernutrition (**Figure** **6**A and Figure S8A‐D), for 26 days. We found that soy peptide supplementation significantly increased weight and rapidly improved WAZ in U‐NHPs compared to soy and whey protein supplementation (Figure 6B). In particular, the 1.5 g kg⁻^1^/day soy peptide which supplies 6.0 kcal kg⁻^1^/day achieves a weight gain velocity of 5.11 g kg⁻^1^/day comparable to the expected weight gain velocity of 5–10 g kg⁻^1^/day when RUTF is used for the treatment of undernutrition in humans.^[^ ^39^ ^]^ Additionally, biochemical parameters including serum albumin, total proteins, TG, BUN, CRP, and globulin levels as well as albumin/globulin ratio were remarkably reversed toward normalcy in soy peptide‐treated U‐NHPs, a result that was not observed in soy‐ and whey protein treatment (Figure 6C‐H; Figure S8E‐M; Table S15). With the above findings, we proceeded to performed pathological examination and transcriptome profiling to uncover the undernutrition alleviation effect of soy peptide in the previously identified impaired organs. Remarkably, the degeneration of splenic white pulp along with expansion of red pulp was reversed (Figure S9A‐C). In addition, there was a trend of decrease in hemosiderin deposition upon soy peptide supplementation (Figure S9D,E, Supporting Information). Regarding the kidney, glomerular area was increased and the previously observed glomerulosclerosis, tubular atrophy, and epithelial vacuolization were also attenuated (Figure S9F,G, Supporting Information), in line with the repudiation of the prior downregulation of RNA splicing and spliceosome pathways (Figure S9H, Supporting Information). In the muscle, more regular nuclei distribution was observed after soy peptide treatment, indicating improved muscle regeneration. Also, muscle fibrosis and fatty infiltration are greatly relieved (Figure S9I,J, Supporting Information). Moreover, muscle contraction‐related pathways were upregulated in U‐NHPs supplemented with soypeptides (Figure S9K, Supporting Information).

**Figure 7:**
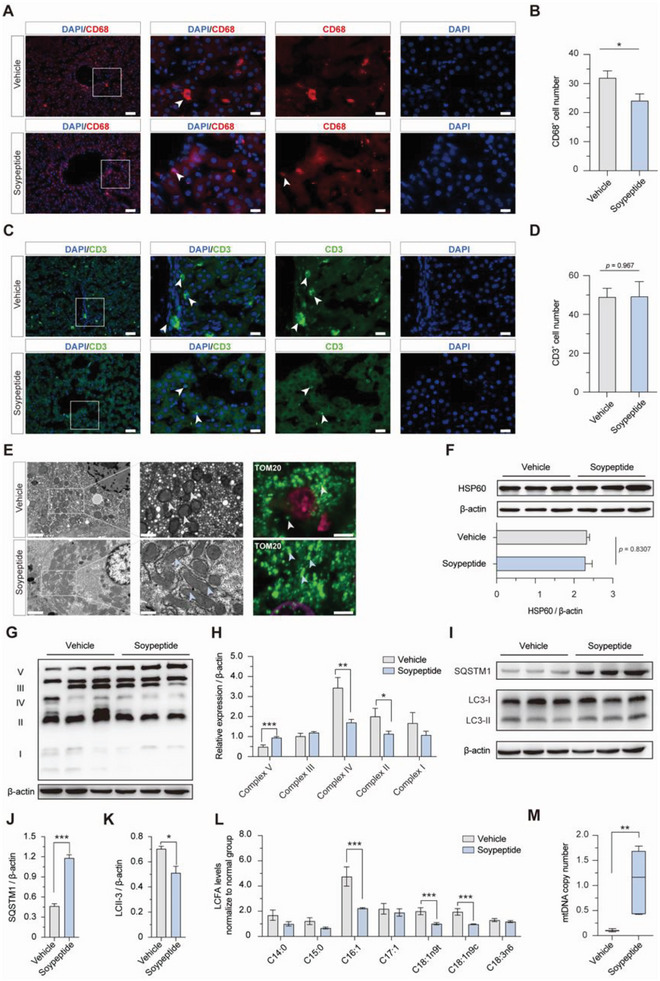
Soy peptide treatment reprogram hepatic fatty acid metabolism to ameliorate steatosis. A) Immunofluorescence staining of CD68^+^ showing activated macrophages. Scale bar: 500 µm (top panel) and 100 µm (bottom panel). B) Relative CD68^+^ count in liver between the N‐NHPs and U‐NHPs. C) Immunofluorescence staining of CD3^+^ showing T cell infiltration in U‐NHP liver. Scale bar: 500 µm (top panel) and 100 µm (bottom panel). D) Relative CD3^+^ number in liver between the N‐NHPs and U‐NHPs. E) TEM of liver periportal hepatocyte showing mitochondrial morphology (Scale bar: 1 µm, left panel; 0.5 µm, right panel) and immunofluorescence staining of mitochondrial outer membrane marker TOM 20 (Scale bar: 100 µm). F) WB and quantification analysis of relative protein levels of HSP60. G) Western Blot analysis of OXPHOS complexes I‐V. H) Quantification of relative OXPHOS complexes I‐V levels. **I)** Western Blot analysis of SQSTM1 (p62) and LC3‐II. J) Quantification of the relative protein levels of p62 between vehicle and soy peptide. K) Quantification of the relative protein levels of LC3‐II between vehicle and soy peptide. L) Comparative analysis of LCFA levels. M) Relative hepatic mtDNA copy number. (n = 6 for each group). Statistical analysis was determined using ANOVA with post hoc Dunnett's test. *P < 0.05; **P < 0.01; ***P < 0.001. U‐NHPs: Undernourished non‐human primates; Normal: N‐NHPs; vehicle: U‐NHPs; soy peptide: soypeptide‐treated U‐NHPs. SFA: Saturated fatty acids; UFA: Unsaturated fatty acids; PUFA: Polyunsaturated fatty acids; MUFA: Monounsaturated fatty acids.

The liver architecture was largely restored after soy peptide treatment, as shown by the amelioration of hepatocyte ballooning, vacuolization and fat droplets along with significant reduction in histological injury degree (Figure 6I,J). Intriguingly, pathways associated with fatty acid derivative binding and fatty‐acyl‐CoA binding were upregulated in the liver (Figure S9L, Supporting Information). Furthermore, Oil Red O staining and triglyceride levels showed that the increased hepatic TG accumulation in U‐NHPs was significantly attenuated (Figure 6K–M). The fuzzy c‐means clustering was then applied to identity intuitive patterns of change in hepatic metabolite accumulation in response to soy peptide supplementation (Figure 6N,O; Figure S9M–P, Supporting Information). Predictably, the clustering showed differential abundance of metabolite between the vehicle, U‐NHP, and the soy peptide‐treated U‐NHPs. Remarkably, in clusters 3 and 6, the undernutrition induced alternation in hepatic metabolite abundance were reversed by soy peptide supplementation toward normal equilibrium levels (Figure 6N,O). More importantly, the abundance of metabolite groups such as lipid and lipid molecules, organic acids, and peptides were restored to comparable N‐NHP levels in U‐NHPs treated with soy peptides, lending merit particularly to the restoration of hepatic lipid metabolism. Moreover, the previously observed disrupted SFA, UFA, PUFA, and MUFA levels were remarkably reversed toward normalcy in soy peptide‐treated U‐NHPs (Figure 6P‐T). Additionally, the ω6/ω3 ratio, an indicator of fatty acid oxidation, decreased in soy peptide supplemented group (Figure 6U). In addition, the macrophage activation in U‐NHP liver was relieved, as shown by the decreased number of CD68^+^ macrophages (**Figure** **7**A‐D), implying significant decline in inflammation. The above results indicated that soy peptide supplementation alleviated hepatic steatosis in U‐NHPs.

The reversal of hepatic mitochondrial dysfunction was revealed by a noticeable recovery of mitochondrial structural integrity as indicated by IF staining of NHP liver (Figure 7E,F). Since mitochondrial dysfunction was found closely associated with OXPHOS perturbation in U‐NHPs with lower complex V and higher II / IV levels, here, we revealed increased complexes V and decreased II and IV levels after soy peptide treatment and also found that, the hydrolysis of OXPHOS complexes was significantly reversed (Figure 7G,H). In addition, soy peptide supplementation has been shown to significantly reverse alterations in LC3‐II and p62 levels in U‐NHPs. This indicates that soy peptides not only neutralize the impacts of undernutrition, but also promote the regulation of autophagic pathways in the liver (Figure 7I–K). Furthermore, a significant decrease in LCFAs (C16:1, C18:1n9t and C18:1n9c) indicated the restoration of mitochondrial function, with significant recovery of mtDNA copy number (*P* < 0.05) (Figure 7L,M).

**Figure 8:**
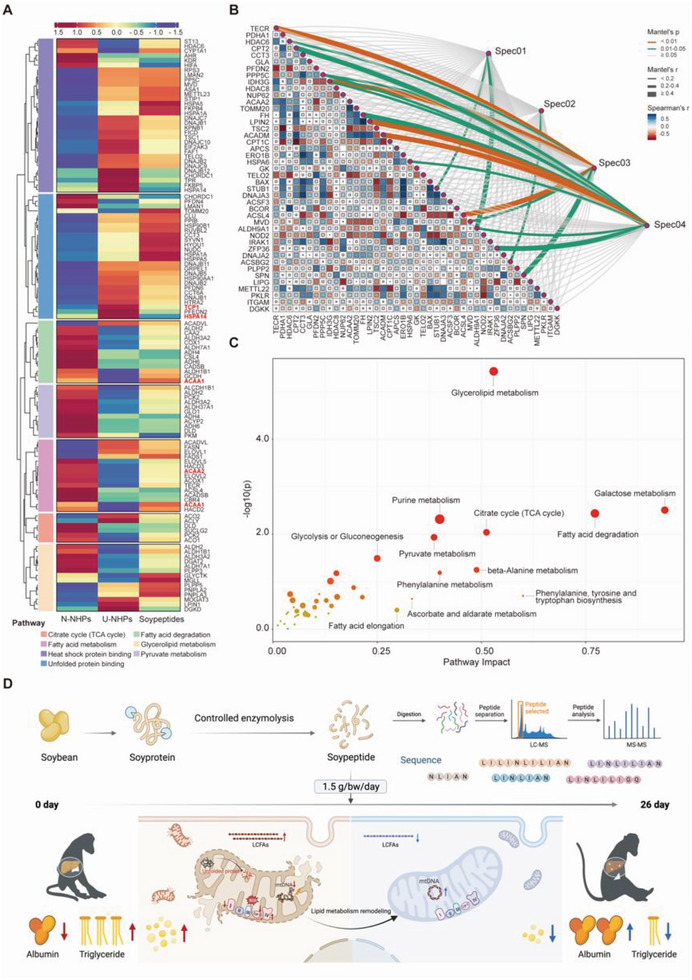
Soy peptide treatment resets hepatic lipid metabolism in U‐NHPs. A) Heatmap showing differential expressed genes between the N‐NHPs, U‐NHPs, and soy peptide‐treated U‐NHPs. B) Inter‐omic analysis of differentially expressed genes and differential metabolites in the liver of soy peptide‐treated U‐NHPS. C) Pathways associated with significant correlated DEGs and metabolites in the liver of soy peptide‐treated U‐NHPs. D) Schematic diagram of the molecular mechanism for the reprograming of hepatic lipid metabolism in U‐NHP livers and attenuation of undernutrition upon treatment with soy peptides.

The expression level of genes involved in fatty acids degradation and mitochondrial stress were mapped among N‐NHPs, U‐NHPs, and soy peptide‐treated U‐NHPs (**Figure** **8**A). Remarkably, the DEGs signature in U‐NHP liver shifted toward normalcy in response to soy peptide treatment, especially genes involved in fatty acid degradation and unfolded protein binding response pathways. Notably, the expression level of genes encoding proteins catalyzing fatty acid β‐oxidation as well as enzymes modulating unfolded protein were upregulated after soy peptide treatment (Figure 8A). Furthermore, joint pathway analysis was performed between hepatic DEGs and differential metabolites to construct a co‐expression network (Figure 8B). Strikingly, glycerolipid metabolism, fatty acid degradation, and galactose metabolism were identified as the most enriched pathways upon soy peptide treatment (Figure 8C), denoting that soy peptide modulates triglycerides degradation in consonance with the Oil Red O staining results.

Taking together, we used controlled enzymatic hydrolysis technique to maximize the diversity of soy peptides and revealed that soy peptides treatment effectively revitalizes mitochondrial function and OXPHOS complexes, thereby reprograming hepatic fatty acid oxidation to relieve hepatic steatosis and mitigated undernutrition in U‐NHPs (Figure 8D).

## 3 Discussion

Undernutrition is a prominent feature of not only inadequate nutrient intake, but also both chronic and acute diseases as well as aging.^[^ ^6^ ^]^ However, despite its widespread prevalence, an in depth understanding of its pathophysiological processes remain opaque.^[^ ^2^, ^6^ ^]^ In this study, we identified undernourished non‐human primates (U‐NHPs) using a novel criterion (WAZ ≤ −1.83) based on WHO‐recommended anthropometric indices. Significant impairments in multiple organs in U‐NHPs were observed, and in particularly, dysregulated liver lipid metabolism due to mitochondrial dysfunction and fatty acid oxidation imbalance. Based on these findings, soy peptides were proven as effective in mitigating undernutrition by modulating hepatic fatty acid metabolism.

Human undernutrition is a systemic disease with complex trait generally accepted to be caused by insufficient food intake or hunger. Other conditions such as HIV, cancer, kidney failure, IBD, and infections leading to severe and recurrent diarrhea and vomiting which either increases energy requirements or impairs nutrient metabolism also substantially predispose to undernourishment in humans. Although the exact cause of undernourishment in our U‐NHP model appears unknown, the monkeys exhibited signs of recurrent diarrhea and significant intestinal inflammation. These could potentially undermine nutrient absorption and utilization thereby predisposing to undernourishment synonymous to certain undernutrition causes of human relevance.^[^ ^2^ ^]^ Irrespective of the underlying causes, acute undernutrition has been associated with signs of atypical metabolism underpinned by nutrient scarcity. Indeed, nutrients and their metabolites not only serve as building blocks of cellular structures and as fuel sources, but also serve as direct modifiers of protein function, potent signaling molecules as well as inducers and repressors of gene expression.^[^ ^40^ ^]^ In line with this, certain amino acids have been shown to act as signaling molecules to regulate cellular growth and proliferation,^[^ ^41^, ^42^ ^]^ implying their scarcity could adversely alter physiological homeostasis. Thus, conditions leading to or exacerbating protein deficiency interfere with metabolic signaling, impair cellular structures, and ultimately disrupts growth. In the end, undernutrition is a metabolic dysregulation condition manifesting in severe wasting phenotypes.^[^ ^15^, ^43^ ^]^ At the organ and systemic level, this is reflected in electrolyte imbalance, hypoalbuminemia, oxidative stress, and hepatic steatosis.^[^ ^2^, ^6^ ^]^ So far, several studies have focused on profiling the serum metabolic profile of undernourished children revealing several dysregulated metabolites.^[^ ^44^ ^]^ Using the U‐NHP model, we show that hepatic lipid metabolism remains the anchored metabolic aberration in undernutrition. Evidence also suggest that energy and metabolic dysregulation could still persist in nutritionally recovered undernourished individuals^[^ ^44^, ^45^ ^]^ indicating that organ level recovering may lag nutritional recovery assessed by weight gain and improved WAZ values. This phenomenon which is believed to underline the high relapse rates^[^ ^9^ ^]^ may be as a result of the inability to comprehensively assess organ recovery including pathological examination in treated acute undernourished subjects.

Tracing back the attempts using rodent models for undernutrition study, weight loss is generally the main threshold for defining undernutrition but this could not adequately recapitulate pathological features of undernutrition.^[^ ^43^ ^]^ For example, mice fed a lower protein diet (<10%) demonstrated significant weight loss implying undernutrition status. However, different levels of low protein feed (e.g., 1% or 5%) caused approximately similar weight loss with contradicting pathological impairments of reduced or enlarged liver (hepatomegaly) respectively.^[^ ^8^, ^32^ ^]^ Thus, it remains unknown what degree of weight loss in rodents could trigger the systemic response and pathological features prototypical of human undernutrition. Moreover, the WHO recommended criteria for diagnosing undernutrition (involving z‐scores such as WAZ) has rarely been used in evaluating animal models. A piglet model established using the z‐score criteria was however found to be incapable of mimicking hypoalbuminemia and increased TG, two of the most critical clinical signs observed in undernourished humans.^[^ ^2^, ^6^, ^10^ ^]^ Typically, the construction of WAZ requires a large sample size to estimate the weight‐for‐age ratio, the measure of healthy growth in the model species. As such, using a total of 1636 *Macaca fascicularis* monkeys, we built a WAZ screening method consistent with the definition of undernutrition. Herein, we have generated the average weight‐for‐age ratio (1.30 ± 0.34 for males; 1.12 ± 0.34 for females) through a rigorous systematic procedure that could serve as a great resource to the field. Based on this, WAZ ≤ −1.83 was derived as the cutoff criteria for identifying undernutrition in *Macaca fascicularis* monkeys, in accordance with the WHO‐recognized definition of human undernutrition.

Undernutrition‐associated organs contributing to or affected by it still remains unclear. Moreover, the fundamental understanding of human undernutrition is greatly hampered by the under‐investigated pathological impairments. So far, autopsy or biopsy from undernourished individuals could not be easily obtained due to the obscure understanding of the preferential organ. Taking advantage of the availability of autopsy from NHPs, we analyzed alterations in liver, muscle, kidney, and spleen using pathological staining, transcriptome, and untargeted metabolome to generate multidimensional signatures of organ impairment in undernutrition. Significantly, hepatic lipid metabolism dysfunction was unearthed as a pivotal metabolic abnormality in U‐NHPs.

As the focal organelles of lipid metabolism under physiological conditions, mitochondrial dysfunction, but unlikely peroxisome dysfunction, was determined as essential for undernutrition‐associated hepatic steatosis in U‐NHPs. By contrast, a prior study in rodents propounded that undernutrition‐related hepatic steatosis involved the simultaneous dysregulation of peroxisomal and mitochondrial function, which might be due to specie differences. In support of this, we found that key genes regulating peroxisome lipid metabolism (ACOx1, PEX2, PEX12, etc.) showed a pattern of lower expression at baseline (normal physiological conditions) in NHPs compared to rodents, suggesting a potential disparate response to these genes under nutrient deprivation between rodents and NHPs. Of particular importance, it was also noted that the expression pattern of these genes differed greatly between rodents and humans but was very similar between NHPs and humans, intimating that lipid dysregulation in NHPs might more closely reflect human undernourished conditions.

Mitochondrial FA β‐oxidation, the major pathway for the degradation of FAs, is tightly linked to the function of OXPHOS. Generally, the electron transport process in complexes I‐IV create an electrochemical proton gradient, which allows for the phosphorylation of ADP to ATP by complex V. In U‐NHPs, increased complexes II and IV whereas decreased complex V were observed. Conversely, most low protein induced‐hepatic steatosis undernutrition rodent models always presented with reduced complex I.^[^ ^8^, ^32^ ^]^ However, the attenuation of hepatic steatosis did not always rescue complex I activity, denoting that impairment in complex I might be a direct result of low protein diet and not the cause or effect of hepatic lipid accumulation. On the contrary, the activity of complexes II, IV, and V in our non‐induced U‐NHP model was restored upon soy peptide treatment. Therefore, we postulate that complex II, IV, and V, may be more closely related with undernutrition‐associated hepatic steatosis. Nevertheless, it remains unclear whether these defects in OXPHOS complexes precede, accompany, or are a direct result of the pathogenesis of undernutrition.

From the observed centrality of mitochondrial dysfunction in hepatic FA β‐oxidation in U‐NHPs, it stands to reason that targeting the restoration of mitochondria functional integrity to re‐establish lipid metabolism homeostasis would be a viable therapeutic option for undernutrition. Current undernutrition therapeutics relies heavily on dietary protein,^[^ ^36^ ^]^ with the WHO recommending that 10–12% of total energy of therapeutic diets must be protein sourced. However, protein exerts its biological function through the peptides and amino acids encrypted in the parent protein which are released upon GI digestion. To this end, WHO propounded that protein quality, which is the digestibility, bioavailability and amount of each individual essential amino acid present in a particular protein, is an indispensable determining factor for the optimal recovery from undernutrition. Aside the moderate improvement in weight (5.11 g kg⁻^1^/day), biochemical readouts and profound restoration of hepatic lipid metabolism in U‐NHPs treated with soy peptides, we observed no significant effect of both soy protein and whey protein treatment on U‐NHPs. Indeed, in malnourished individuals with significantly poor digestion and absorption capacities and general impaired gut function,^[^ ^46^ ^]^ proteins may not only fail to be efficiently converted, but may also pose significant burden to the digestive system. Moreover, proteins especially plant‐derived, are predominantly compact globulins, alcohol‐soluble proteins and glutens that hinder their hydrolysis into small peptides and amino acids, ultimately resulting in low bioavailability.^[^ ^47^ ^]^ Additionally, the anti‐nutritive component, saponins, present in plant‐based proteins can result in disruption of tight junction and oxidative damage in the distal intestine.^[^ ^48^ ^]^ Fortunately, the enzymatic hydrolysis of soy protein into peptides has been shown to effectively reduce the levels of anti‐nutrient factors, including trypsin inhibitors, lectins, and allergenic proteins.^[^ ^49^ ^]^ Furthermore, the use of peptide‐based enteral formulas in individuals with comprised gastrointestinal function have been associated with improved GI tolerance, better nitrogen retention, reduced diarrhea and restoration of gut integrity compared to free amino acids and whole‐protein formulas.^[^ ^50^ ^]^ In accordance with this, we recently proved that soy peptide supplementation is more efficacious in alleviating *E. coli*‐induced septic inflammation compared to amino acid mix in NHPs.^[^ ^51^ ^]^ In addition to low molecular weight, peptides have high solubility, low viscosity and low allergenicity which confers high nutritional value through improved absorption. The distinct structural features of proteins and peptides result in their disparate digestive and absorptive properties. Fractionation of soy peptide have been shown to yield high amounts of branched chain amino acids (BCAA)‐rich di‐ and tripeptides which have been shown to have a high capacity for being transported across the epithelial cells of the small intestines into cells by peptide transporters (PepT1).^[^ ^52^ ^]^ In a landmark study, authors showed that soy peptide intake resulted in a rapid increase in serum concentrations of essential, BCAA and aromatic amino acids compared to consumption of soy protein and equivalent amounts of amino acids mixture in healthy men, indicating a faster and more efficient absorption of soy peptide compared to proteins.^[^ ^53^ ^]^ The quicker release and absorption of these essential protein building blocks from the intake of soypeptide suggest they could reach target organs swiftly and also ensure rapid protein synthesis for bodily use. In animal husbandry, piglets fed fermented soy peptides exhibited superior protein and energy utilization compared to soy protein.^[^ ^54^ ^]^ An earlier study further proved that soy peptides could alter lipid metabolism by decreasing the secretion of apolipoprotein B‐100‐containing lipoproteins while increasing the mRNA of genes related to beta‐oxidation of fatty acids.^[^ ^55^ ^]^ These could underline the relative increase in weight, improvement in biochemistry readouts and the modulation of hepatic lipid metabolism in soy peptide compared with soy and whey protein‐treated U‐NHPs.

Additionally, hepatic mitochondrial morphology was repaired clearly demonstrating the restoration of the structural and functional integrity of the mitochondria. Moreover, the disruption of OXPHOS complexes (II, VI, and V) was reversed and stabilized in U‐NHPs treated with soy peptides. Prior study revealed that the restoration of OXPHOS complexes could be achieved by enhancing mitochondrial function via the reduction of ROS generation.^[^ ^56^ ^]^ This implied that the soy peptides could restore redox homeostasis to revive mitochondrial function which may also account for the stability of OXPHOS complexes. The restoration of mitochondrial function then reprogramed hepatic fatty acid oxidation to relieve hepatic steatosis and attenuated undernutrition.

Contrary to current treatment therapies which focuses on rapid weight gain by principally supplying energy to relieve undernutrition phenotype, our novel strategy using soy peptide supplementation mechanistically targets hepatic lipid metabolism to mitigate undernutrition. This could potentially promote community‐based management of cases and make treatment economical by sourcing the vital treatment resource from the local economy.

In spite of the remarkably findings demonstrated here, the current work comes with some limitations. First, even though we observed significant diarrhea and inflammation in the U‐NHPs, the exact causes of failure to thrive remains unknown. However, the presence of diarrhea and intestinal inflammation which are common in human undernourishment makes the U‐NHP model of human relevance. Moreover, the findings in here remain preliminary as such the study is short of functional experiments to determine the mechanism by which soy peptide mitigate hepatic lipid metabolism in undernutrition, which is currently ongoing.

## 4 Experimental Section

### Ethics Statement

All animal experimental protocols in this study were reviewed and approved by the Institutional Animal Care and Use Committee of Huazhen Biosciences (Guangzhou, China), under the title “Beneficial effect of food‐derived protein and peptide in undernourished *Macaca fascicularis* monkeys” (#HZ2020074) (Appendix 1 and 2). With Huazhen Biosciences (Guangzhou, China) as the research subsidiary of the AAALAC‐certified animal breeding center (Appendix 3), Guangzhou Aojun Biological Technology Co., Ltd. (https://www.aaalac.org/), the study adhered to principles stated in the Guide for the Care and Use of Laboratory Animals, Cynomolgus non‐human primate (NHP) monkeys (*Macaca fascicularis*).

### Monkey Housing and Feeding

*Macaca fascicularis (M. fascicularis)* monkeys were being housed at the Animal Experimental Center of the Huazhen Animal Breeding Centre (113.52°E, 23.63°N, Conghua, Guangzhou, China), which is a subsidiary of an AAALAC‐accredited animal research facility, Guangzhou Aojun Biological Technology Co., Ltd (Appendix 3). All monkeys used were negative for simian retrovirus, SIV, and simian T‐lymphotropic virus. Animals were housed in standard indoor cages with normal environmental conditions. All monkeys were being fed a standard monkey chow obtained from Guangzhou Guolong Technology Co, Ltd. (Guangzhou, China). The feeding regime was pellet feed for breakfast at 8:30 am. and dinner at 16:30 pm. daily. All animals were also served fruits as lunch at 12:30 noon every day. In addition, all animals were allowed access to water ad libitum. All edibles and drinkables meant for animals were periodically screened for microbial presence and other contaminants so as not to interfere with the study and the health of the animals.

### Determination of Body Condition Scores (BCS)

Body condition scoring was determined according to the previously report method.^[^ ^57^ ^]^ Briefly, NHPs were fasted for 12 h before BCS estimation, which required sedation with ketamine (10 mg k^−1^g IM; Fort Dodge Animal Health, Fort Dodge, IA). The sedated animal was then subjected to gentle palpation of several body sections to assess bone prominence, the presence of a subcutaneous fat layer, and muscle mass. Palpations typically included of hips/pelvis which covers wings of the ilium, sacrum, and ischium, the spine involving the lumbar and thoracic spine regions for prominence of spinous processes, presence of subcutaneous fat layer, and epaxial musculature. Subsequently, the thorax was also palpated over the rib cage and scapulae aimed at assessing the prominence of the ribs and the presence of subcutaneous fat. Muscle mass was assessed in association with the palpation of the bony prominences of the hips, spine, and scapulae. The quadriceps, hamstrings, biceps, or triceps were further assessed for additional muscle masses. Other bodily areas assessed were the head and the abdomen.

### BCS Scoring

The nutritional status was assessed using the BCS scoring system (*Macaca mulatta*) which uses a scale comprising half units that become available starting in 2005 and ranged from 1 to 5 with a total of 9 grades, lower values represent emaciated to lean conditions (1 to 2).^[^ ^57^ ^]^ In the current study, the selection and allocation into the normal or non‐thriving group was based on the BCS. A total of 1636 *Macaca fascicularis* monkeys from an indoor‐housed breeding colony were evaluated by the veterinary staff and assigned to one of 9 BCS score groups. To prevent bias and false classification, the BCS score for each *Macaca fascicularis* monkeys required the average score given by three experienced veterinarians. Monkeys with BCS < 2 were classified into the non‐thriving group while those with BCS ≥ 2 were classified into the normal group.

### Anthropometric Measurements

All anthropometric indices were measured by qualified veterinarians and measured at scheduled time points.

### Weight

To measure the weight of monkeys, the weight of a member of the veterinarian team was measured to the nearest 0.1 kg as *W_1_*, using a Tanita MC‐780A scale with the veterinarian wearing light clothes and no shoes. The veterinarian then carried the monkey in his arms and weighed again (the obtained weight as *W_2_*) following the same procedure as previously. The weight of the monkey was then calculated as *W*  =  *W*_2_ − *W*_1_.

### Mid‐Upper Arm Circumference (MUAC)

MUAC was measured on the left arm to the nearest 0.1 cm using a MUAC tape. The measurement was taken as the circumference around the mid‐point of the distance between the tip of the elbow (the olecranon process) and the tip of the shoulder (the acromial process), from the posterior aspect of the arm. The tape was kept straight touching the skin ensuring that it does not compress the skin or tissue.

### Head Circumference

Head circumference was measured to the nearest 0.1 cm using an un‐stretchable tape measure. The tape was securely wrapped around the widest possible circumference of the head covering the broadest part of the forehead above the eyebrow, above the ears, and the most prominent part of the back of the head.

### Skinfold Thickness

A skinfold caliper was used to measure the thickness of the pleats at subscapular and triceps skinfolds. To measure the skinfold at these locations, the forceps were held with the right hand of veterinarian. The skin and subcutaneous tissue of the measuring part were then firmly grasped with the left hand along the long axis of the body to fully separate the skin from the muscles below using the thumb and the index finger. The contact surface of the calipers was directly placed below the lifted and kneaded part at 90°. The subscapular and triceps measurements were done on the left side of the body. Measurements were taken in triplicates and the mean recorded as the skin fold for a particular sight for each monkey.

### Determination of Weight‐for‐Age z‐Score (WAZ)

To calculate the WAZ, 1387 monkeys with BCS ≥ 2 were sampled from a total of 1636 and used as the reference cohort (857 males and 530 females). The weight and age of the monkeys were used to calculate the mean and standard deviation of weight‐for‐age ratio based on gender to obtain the reference weight distribution. To define undernutrition in the monkeys, 249 non‐thriving monkeys (BCS < 2) were selected and paired in an approximately 1:2 ratio with 454 normally growing monkeys (BCS ≥ 2) as the study cohort. Then, the z‐score was calculated by the standard formula as follows:

(1) $$z-score=\frac{X-\;\mu }{\sigma }$$

Where, X is the body weight of each monkey, µ and σ are the mean weight‐for‐age ratio and standard variance of the reference population respectively.

### Serum Biochemical Analysis

Blood samples were collected by vacutainer and then separated by centrifugation at 3500 rpm for 15 min at 4 °C. The serum samples (200–300 µL) were immediately analyzed by the Cobas C311 analyzer device (Roche, Switzerland).

### Tissue Acquisition

After overnight fasting, the monkeys were anesthetized with ketamine hydrochloride (10 mg k^−1^g) via intramuscular injection, and then deeply anesthetized with pentobarbital sodium (100 mg k^−1^g) by intravenous injection. Two professional veterinarians determined the sacrifice of animals by judging if breathing and heartbeat were completely stopped, and there was no nerve reflex and the muscles of arms and legs are relaxed. Then, the liver, spleen, kidney, and muscle (right lower limb) were excised as well as cecal content, into a snap‐frozen in liquid nitrogen, and stored at −80 °C until further analysis. More importantly, the animal carcasses were disposed of harmlessly.

### Histology and Pathology Analysis

Monkey tissues were fixed in 4% paraformaldehyde (PFA) overnight at 4 °C and embedded in paraffin or optimal cutting temperature compound (OCT) before sectioning. Sections were stained with H&E or Oil Red O and photographed using a DM 6 microscope (Leica, Germany). For liver lipid staining, we used freshly cut cryostat sections (10 µm) which were then defrosted and air‐dried for 10 min. The areas around the tissue sections were traced with a liquid blocker pen, and the sections were incubated in a solution of 3.7 mg mL⁻^1^ Oil Red O in 60% isopropanol for 10 min. Subsequently, the sections were rinsed under running tap water for 30 min, mounted with Clear‐Mount, covered with coverslips, allowed to set for 10 min at room temperature, and finally, the coverslip edges were sealed with nail polish to create an airtight seal. This staining procedure was repeated at least twice to confirm the consistency of staining between batches.^[^ ^58^ ^]^ Images were processed by Image Pro plus (6.0) software. Quantitative analysis was performed according to the previously reported by Adam et, al.^[^ ^59^ ^]^ and Tang et, al.^[^ ^60^ ^]^

### Triglyceride Measurement and Analysis

Triglyceride measurements were determined from liver supernatants using the Amplex Red Triglyceride Assay Kit (Beyotime, Shanghai, China). Triglyceride levels were first normalized to starting tissue weight and then compared against N‐NHP or U‐NHP samples.

### Transmission Electron Microscopy

Monkey tissues were fixed in 2.5% glutaraldehyde solution at 4 ° C for 2–4 h. Samples were then imaged by transmission electron microscopy (Hitachi H‐7650, Tokyo, Japan).

### Immunofluorescent Staining

Monkey tissues were fixed in 4% PFA overnight at 4 °C. Immunofluorescent staining on frozen liver tissue sections of 8 µm thickness. Tissue sections were fixed at 4% PFA at room temperature for 30 min. Staining for CD68^+^ (macrophage marker), CD3^+^ (T cell marker), PEX14 (peroxisomal marker), and TOM20 (mitochondrial marker) was performed according to a standardized protocol for immunofluorescence on frozen tissues. Tissue slices were then rehydrated with PBS, blocked with 10% serum albumin in PBST for 1 h at room temperature (RT) and incubated with primary antibodies (diluted in PBST with 1% serum albumin) overnight at 4 °C. Subsequently, these sections were stained with secondary antibody (1:500) at room temperature for 2 h. Then sections were stained with DAPI for 5 min and then mounted with 60% glycerol. Stained brain tissues were observed and captured with a Zeiss LSM 880 confocal microscope (Carl Zeiss, Germany). Quantification analysis of the fluorescent intensity was by the ImageJ software.

### Immunoblotting Analysis

Immunoblotting 100 mg liver samples were homogenized in 1000 µL ice cold RIPA (Dingguo Changsheng, Beijing, China) lysis buffer containing PMSF (1:100) (Dingguo Changsheng, Beijing, China), and protease Inhibitor Cocktail (EDTA‐Free, 100X in DMSO) (1:10) (Bimake, Houston, TX, USA). Homogenates were solubilized for 2 h at 4 °C and centrifuged at 13000 × g for 15 min at 4 °C. Protein concentrations in the supernatants were determined using the BCA protein assay kit (Dingguo Changsheng). Equal amounts of protein in liver tissue lysate (10 µg) were resolved with Mini‐PROTEAN^®^ Tetra Handcast Systems (Bio‐Rad) (10–15% gels) at 120 V for 80 min. Proteins were transferred to the PVDF membrane (Bio‐Rad) using Mini Trans‐Blot® Cell (Bio‐Rad). Membranes were blocked with 5% bovine serum albumin (BSA) in a Tris‐buffered saline (TBS) containing 0.1% tween 20 (TBST) for 2 h. The membranes containing liver tissue proteins were incubated overnight at 4 °C with primary anti‐PMP70 antibody produced in rabbit (1:1000, Sigma‐Aldrich, p0497‐200UL), anti‐HSP60 antibody (1:1000, Abcam, ab46798), β‐actin loading control antibody (1:1000, Invitrogen, MA5‐15739), and Total OXPHOS Rodent WB Antibody Cocktail (1:5000, Abcam, ab110413). Next, membranes were washed 3 × 10 min with TBST and incubated with a corresponding horse‐radish peroxidase‐conjugated secondary antibody for 2 h at RT. Then a final wash of 3 × 10 min with TBST. Subsequently, immune complexes were detected using the ECL kit. The membrane was scanned for the relative value of protein expression using the TANON 6600 Luminescent Imaging Workstation (Tanon Science & Technology Co., Ltd., Shanghai, China), measuring the grayscale with Image‐Pro Plus software, version 6.0 (Media Cybernetics, Inc., Rockville, MD, USA).

### Hepatic Fatty Acid Profiling

Liver fatty acid profiling was according to the previously reported by Bauer et, al.^[^ ^25^ ^]^ with modifications. Liver tissues were excised and immediately kept on dry ice and stored at −80 °C until usage. For profiling, 50 mg sample was homogenized (Ultra‐Tarrax homogenizer, IKA, Staufen, Germany) at 4 °C in a homogenization buffer (1 µM 2,6‐di‐*tert*‐butyl‐4‐methylphenol, 1 mM diethylenetriamine penta‐acetic acid, 2 mM ethylenediamine tetra‐acetic acid, 5 mM 3‐(*N*‐morpholino) propanesulfonic acid, with 180 mM potassium chloride, and adjusted to pH 7.4). Samples were normalized by protein content. Total hepatic lipids of the homogenates were extracted using chloroform‐methanol (2:1 [v/v]; 3 times) with 0.01% butylated hydroxytoluene. The chloroform was then eliminated with nitrogen and the FAs were transesterified by incubation using 2.0 mL 0.5 mol L⁻^1^ sodium‐methanol (2 g/100 mL, w/v) buffer at 75 °C for 15 min followed by adding BF_3_‐methanol (1:2, v/v) solution. Following transesterification, *n*‐hexane (2.0 mL) and saturated NaCl (1.0 mL) were added for extraction of FAMEs. After thoroughly vertexing, the mixture was allowed to settle down for 30 min at 4 °C subsequently evaporated under nitrogen gas, and then re‐dissolved in *n*‐hexane. Then, 1 µL was used for subsequent gas chromatography‐mass spectrometry (GC‐MS/MS) analysis.

### Metabolome Analysis

About 25 mg of the liver sample was harvested into a 1.5 mL Eppendorf tube, and 800 µL of extraction solution (methanol: acetonitrile: water = 2:2:1, v:v:v, pre‐cooled at −20 °C) and 10 µL of internal standard, then add two small steel balls were mixed with the tissues for grinding (50 Hz, 5 min), ultrasonicate in a 4 °C water bath for 10 min, and then stored in a −20 °C refrigerator for at least 1 h. After 15 min centrifugation (25 000 rpm), 600 µL of the supernatant was transferred into a freezer vacuum concentrator for, added 200 µL of reconstitution solution (methanol: H_2_O = 1:9, v:v) for reconstitution, vortex for 1 min, and then ultrasonicated for 10 min in a water bath at 4 °C. Centrifuge at 25 000 rpm for 15 min at 4 °C, and placed the supernatant into a loading bottle. A Waters 2D UPLC (waters, USA) tandem Q Exactive HF high‐resolution mass spectrometer (Thermo Fisher Scientific, USA) was used for separation and detection of the metabolites.

### GC‐MS Analysis

Elucidation of liver fatty acid profiling was carried out on an Agilent 8890‐7000D GC‐MS system (Agilent Technologies, USA) fitted with an Agilent 19091N‐133 HP‐INNOWax (30 m × 250 µm × 0.25 µm) column and an Agilent triple‐axis HED‐EM detector. The injection port and the detector were set at 220 °C and 250 °C respectively and the spitless mode was used for the injection. Helium (99.99%) which was used as the carrier gas was set at a flow rate of 2.25 mL min^−1^. The temperature programing employed in the analysis was 5 min at 100 °C, then increased by 5 °C/min to 250 °C, and then maintained at 250 °C for 10 min. The detection time was 45 min. FAMEs were characterized and identified by comparing with original standards (CRM47885, Merck Life Science (Shanghai) Co. Ltd., Shanghai, China). The obtained results were expressed and presented as percent moles normalized against the controls.

### RNA Sequencing—RNA Extraction, Library Construction, Sequencing, and Quality Control

Total RNA was extracted from tissues using Trizol (Invitrogen, Carlsbad, CA, USA) according to the manual instruction. In brief, about 60 mg of tissues were used to extract total RNA. Trizol lyzed tissue was mixed with chloroform/isoamyl alcohol (24:1) in a new tub. After precipitated with isopropyl alcohol and then centrifuged at 13 600 rpm for 20 min at 4 °C. The RNA pellet was washed and dissolved with 25–100 µL of RNase‐free water. Subsequently, total RNA was qualified and quantified using a Nano Drop and Agilent 2100 bioanalyzer (Thermo Fisher Scientific, MA, USA). Oligo(dT)‐attached magnetic beads purified mRNA was fragmented into small pieces and then generated cDNA by the random hexamer‐primed reverse transcription. Afterward, A‐Tailing Mix and RNA Index Adapters were added by incubating to end repair. The cDNA fragments obtained from the previous step were amplified by PCR, and products were purified by Ampure XP Beads, then dissolved in EB solution. The product was validated on the Agilent Technologies 2100 bioanalyzer for quality control. The double stranded PCR products from the previous step were heat‐denatured and circularized by the splint oligo sequence to get the final library. The final library was amplified with phi29 to make DNA nanoball (DNB) which had more than 300 copies of one molecular, DNBs were loaded into the patterned nanoarray and single end 50 bases reads were generated on BGIseq2000 platform (BGI‐Shenzhen, China).

After filtering with FastQC, clean reads were aligned to *M. fascicularis* monkeys v 5.0 retrieved from NCBI genomic database by using the subread aligner (The Subread aligner 2.0.3). The total gene annotated was 53934 which was made up of 20110 protein coding genes, 11790 long noncoding RNAs (lncRNA's) as well as 12648 pseudogenes. Expression levels were produced at the gene level in Reads Per Kilobase of transcript per Million mapped reads (RPKM units) (controlling for gene length and sequencing depth) using featureCounts of Subreads (v 2.0.3). Lowly expressed genes were filtered, and the expression level was converted to transcripts per kilobase million (TPM) data and were log2‐transformed (log2TPM) for subsequent analysis, which showed more comparability between samples.

### RNA Sequencing—Differential Gene Expression Across Tissue

Tissues with at least three samples of each phenotype were analyzed. Principal Component analysis (PCA) was performed to assess sample variability and visualize transcriptome differences between samples. Differential expression was performed with limma. We used the false discovery rate (FDR) < 0.05 and |log twofold change (FC)| > 1.0 as cut‐off for statistical significance in limma analysis. Differentially expressed genes among the liver, muscle, and kidney tissues between the undernutrition and normal phenotype monkeys was then obtained. Differentially expressed genes were then used for Kyoto Encyclopedia of Genes and Genomes (KEGG) and Gene Ontology (GO) over representation enrichment analysis by the clusterProfiler package. To further compare the pathway activity across samples, Gene Set Enrichment Analysis (GSEA) was performed as well. The threshold of GSEA was set at |Normalized Enrichment Score (NES)| > 1 and adjusted *P* < 0.05.

### RNA Sequencing—Linear Regression Analysis and Linear Mixed Model (LMM)

All linear regression analysis and Analysis of Variance (ANOVA) were performed using the LMM and the ANOVA functions in R. LMM was used to assess the contribution of tissue and individual variance to gene expression variation by the lmer4 R package. The restricted maximum likelihood (REML) estimators for the random effects of tissue, individual and residual variance by their sum.

### RNA Sequencing—Cross‐Species Gene and Metabolic Pathway Analysis under NAFLD Phenotype in Three Species

Monkey RNA‐seq data was from this study. Human data was obtained from GEO's GSE160016, which included liver samples from organ donors after cardiac death (DCD; 6 non‐NAFLD donors, 5 NAFLD donors) for liver transcriptome analysis. Mouse data from GEO's GSE222935 and GSE52748 were used to illustrate differential gene expression in NAFLD mice. For the comparison of the gene expression of the three species under normal conditions, TPM values were used as the standard. The formula for converting counts to TPM is as follows:

(2) $$\mathrm{TPM}=\frac{N_{i}/L_{i}\times 10^{6}}{sum\left(N1/L1+N2/L2+\cdots N_{n}/L_{n}\right)}$$

GO and KEGG analyzes were then performed using the methods described above in experimental section.

### RNA Sequencing—Correlation Analysis of Differential Expressed Genes and Differential Metabolites

Spearman correlation analysis was applied to demonstrate the correlation between differential expressed genes and differential metabolites, and *P* < 0.05 was considered statistically significant. The joint pathway analysis was performed by using MetaboAnalyst (https://www.metaboanalyst.ca). The differential expressed genes involved in targeted pathways and all differential metabolites were subjected to the analysis, and the KEGG database was used to perform the enrichment analysis. The most relevant pathways were identified by combining results from powerful pathway enrichment analysis with pathway topology analysis.

### Peptide Affect Lipid Metabolism in Human Liver Organoid System

Human liver organoid model established according to our previously published method^[^ ^61^, ^62^ ^]^ with slight modification. Briefly, hepatocytes were isolated from liver tissues by collagenase digestion and stereoscopic culture of liver organoids was performed. The medium was removed and the differentiated liver organoid culture was washed three times with HBSS (Hank's balanced salt solution). The experimental group was treated with 2.5 mM peptide or amino acid (dissolved in the culture medium). Non‐treated human liver organoids were used as the blank control. After incubating for 24 h, the supernatant was removed, and the cells were washed three times with HBSS. RNA was isolated with TRIzol (Thermo Fisher), with genomic DNA removed by Dnase I. Quality and quantity were assessed via NanoDrop (BioRad) and Bioanalyzer 2100 (Agilent). A TruSeq™ RNA Kit (Illumina) was used for library preparation, followed by sequencing on an Illumina/Hiseq‐2000. Data analysis was conducted in R using DESeq2 for sequencing data, PCA for variability, and Corrplot for correlations.

### Computer Simulation of Hydrolysis of Peptides from Different Food Sources and Cluster Analysis

This method involves using different enzymes in the digestive system to cleave protein sequences from different food sources (plant protein, animal protein, milk, and egg sources). The proteins are cleaved using different enzymes (Pepsin, α‐chymotrypsin, Trypsin, Pancreatic elastase, Plyl oligopeptidase, ThiM oligopeptidase). The proteins from different sources are identified using the protein numbers in Uniprot, and the resulting peptide sequences and their quantities after simulated hydrolysis are used as enzymatic characteristics of the protein. Based on the GibbsCluster algorithm,^[^ ^63^ ^]^ the proteins are divided into four categories based on the peptide sequences and quantities resulting from enzymatic cleavage. Each protein corresponds to different enzymatic hydrolysis result categories for different enzymes. These categories are used as features for each protein, and after dimensionality reduction using PCA, the proteins are clustered using k‐means algorithm.^[^ ^64^ ^]^

### Molecular Descriptor Properties of Peptides

Soybean peptide sequences were obtained from the hydrolysis experiments and structure identification. Peptide sequences with different functions from other sources were obtained from BioPepDB (zju.edu.cn), and the two were combined to form the baseline dataset. The RDkit library in Python was used to calculate the structural descriptors of the peptides. Peptide sequences were used as identification IDs, and peptide descriptors were used as features. The data was standardized, and the dataset was divided into training and validation sets in a ratio of 3:7. PCA was used for dimensionality reduction. SVM is an efficient classification model that has the advantage of dealing with small sample sizes. The Scikit‐learn SVM model was used to predict the functions of soybean peptides obtained from experiments.^[^ ^65^ ^]^

### Soypeptide Preparation by Controlled Partial Enzymatic Hydrolysis

The preparation of soy peptide mix was as previously reported with slight modifications.^[^ ^34^ ^]^ In brief, soybean protein isolate (SPI) was suspended in distilled water at a final concentration of 12.5% (w/w), was then hydrolyzed using Alcalase (Novozymes) and Flavourzyme 500 (Novozymes) in 3:1 ratio at an enzyme‐to‐substrate ratio (E:S) of 1% (w/w). This reaction proceeded for 4 h at 55  °C ± 0.5 with agitation in a water bath, maintaining a constant pH of 6.0 using 1 mol L⁻^1^ NaOH. The process was completed by heating the mixture to 98 °C ± 0.5 for 10 min to terminate the reaction. The filtrate from enzymatic hydrolysates was collected and concentrated by using tangential flow system (BONA‐GM‐T01/57MX, BONA, Shandong, China) with a hollow fiber polysulfone ultrafiltration membrane module (UFP‐5‐C‐35; 5 kDa MWCO, active area of 1.35 m^2^; cytiva, USA), and the concentrated filtrate was further desalted by nanofiltration. Nanofiltration experiments were composed of a thin film composite membrane, from DuraFoul NF8040, with a MWCO of 150–450 Da (SUEZ, USA) The obtained filtrate was then spray‐dried to obtain soy peptide powder for further use.

### Amino Acid Detection

Soy protein and soy peptide samples of 10 mg were digested into 10 mL of 6 mol L⁻^1^ HCl, then dried and reconstituted in 1 mL of 0.02 mol L⁻^1^ HCl. The digested samples were filtered through a 0.22 µm Supro membrane disc filter. Amino acids were measured and quantified using an Amino Acid Analyzer L‐8900 (Hitachi, Japan).

### In Vitro Validation of Steatosis‐Attenuation Capacity of Soy Peptide

LO2 cells in logarithmic growth phase were seeded in well plates and cultured in a cell incubator at 37 °C for 24 h. The culture medium was discarded, and new culture medium was added and further cultured for 24 h. Pre‐heated 10% BSA‐PBS solution and fatty acid mixture solution were added to the culture medium in proportion. Three Experimental groups were set up. (1) Control group: LO2 cells without fatty acid and soybean peptide; (2) Model group: fatty acid (0.8 mM) treated LO2 cells; (3) Soy peptide group: fatty acid (0.8 mM) and Soypeptide (0.1 mg mL⁻^1^) treated LO2 cells. At the end of the culture, the culture medium was removed, and the cells were washed 2–3 times with precooled PBS. The plates were fixed with the addition of 4% paraformaldehyde for 1 h and washed twice with PBS. The cells were then stained with freshly prepared Oil Red O staining solution (Sigma‐Aldrich) for 30 min. The cells were washed three times with PBS to adequately remove any remaining oil red in the well plates. Red lipid droplets distribution in cells was then observed using a microscope (all samples were photographed under the same parameters and light source).

### Experimental Design—Sample Size Determination

The sample size for the treatment was determined using the group comparison‐one‐way ANOVA method of the “resource equation” approach.^[^ ^18^ ^]^ Four (4) test groups vehicle, soy protein, whey protein, and soy peptide were set up. The sample size was calculated according to the formula below:

(3) $$n=\frac{DF}{k}+1$$

where n = number of subjects per group, DF = Degree of freedom, and k = number of groups. Based on the acceptable DFs of 10 (minimum) and 20 (maximum) and the number of test groups of 4 (vehicle, soy protein, whey protein and soypeptide), 4 and 6 U‐NHPs were derived as the minimum and maximum sample size per group respectively. With the knowledge of the high death/casualty rate in the undernourished group of NHPs, we provide for a 20% potential casualty and loss, and capped the sample size to 8 U‐NHPs per test group.

### Experimental Design—Treatment of U‐NHPs

Undernourished NHPs (U‐NHPs) were randomly put into four groups namely, vehicle (average age = 4.3 years and WAZ = −2.23), whey protein treatment (average age = 3.9 years and WAZ = −2.20), soy protein treatment (average age = 4.4 years and WAZ = −2.35), and soy peptide treatment (average age = 4.3 years and WAZ = −2.49). Each group comprised of eight monkeys with no significant difference in gender. In addition to the standard daily feed ration (section 4.2 above), animals in three groups received whey protein, soy protein, and soy peptide, respectively (1.5 g kg⁻^1^/day providing 6.0 kcal kg⁻^1^/day). The protein and peptide powders were each dissolved in 25 mL water and administered by gavage. Accordingly, animals in the vehicle group were treated with 25 mL of saline water also by gavage as vehicle controls (n = 8). All treatments lasted for 26 days.

### Statistical Analyses

In this study, all statistical analysis were performed in R (version 4.3.1) unless otherwise stated. Continued variable data were presented as Mean ± SEM in figures and text. Statistical significance for Welch Modified Two‐Sample t‐test and post‐hoc comparisons are expressed as *P < 0.05, **P < 0.01, ***P < 0.001. Clustering analysis with fuzzy c‐means algorithm was conducted to identify pattern changes in serum metabolites under different interventions.^[^ ^66^ ^]^ FDR adjusted *P* < 0.05 was considered statistically significant in Spearman correlation analysis. Figures for the obtained results were generated using either GraphPad Software (version 8.3.0), adobe illustrator (2022) or the scientific image and illustration software, biorender.com.

The *M. fascicularis* RNA sequencing has been deposited at NCBI under the accession numbers PRJNA797564.

## Conflict of Interest

The authors declare no conflict of interest.

## Author Contributions

Z.X., W.K.A., Z.R., Y.X., W.L., and C.G. contributed equally to this work. Conceptualization was done by J.R., M.Y., and W.L. Chemical synthesis was carried out by Y.X., Z.Z., Z.X., and L.G. Biology experiments were carried out by Z.X., Z.R., Y.X., M.Z., C.Q., and J.T. Methodology, investigation and formal analysis were carried out by W.K.A., C.G., Z.Y., J.Z., M.Y., W.L., C.H., and J.Z. The original draft was written by W.K.A., Z.X., Y.X., C.G., Z.R., W.L., M.Y., and J.R. Review and editing of the draft were conducted by W.K.A., Z.X., Y.X., C.G., Z.R., W.L., M.Y., J.R., M.W., and L.G. Data curation was carried out by Z.Y, M.Y., J.R., X.X, and G.L. Visualization was carried out by C.W., M.W. W.L., C.W., M.Y., and J.R. supervised the project. Funding and resources were acquired by J.R.

## Supporting information

Supporting Information

Supporting Information Table 1

Supporting Information Appendix 1

Supporting Information Appendix 2

Supporting Information Appendix 3

## Data Availability

The data that support the findings of this study are available in the supplementary material of this article.
