# Supplementary material for: Soy Peptide Supplementation Mitigates Undernutrition through Reprogramming Hepatic Metabolism in a Novel Undernourished Non‐Human Primate Model
Source: Adv Sci (Weinh). 2024 May 30;11(29):2306890. doi: 10.1002/advs.202306890 (PMC11304262; doi:10.1002/advs.202306890)
Supplement: Supplementary file 1 — Supporting Information [file ADVS-11-2306890-s004.docx]

Supporting Information

**Soypeptide supplementation mitigates undernutrition through reprogramming hepatic metabolism in a novel undernourished non-human primate model**

*Zhenzhen Xu†, William Kwame Amakye†, Zhengyu Ren†, Yongzhao Xu†, Wei Liu†, Congcong Gong†, Chiwai Wong, Li Gao, Zikuan Zhao, Min Wang, Tao Yan, Zhiming Ye, Jun Zhong, Chuanli Hou, Miao Zhao, Can Qiu, Jieqiong Tan, Xin Xu, Guoyan Liu, Maojin Yao, Jiaoyan Ren**

**
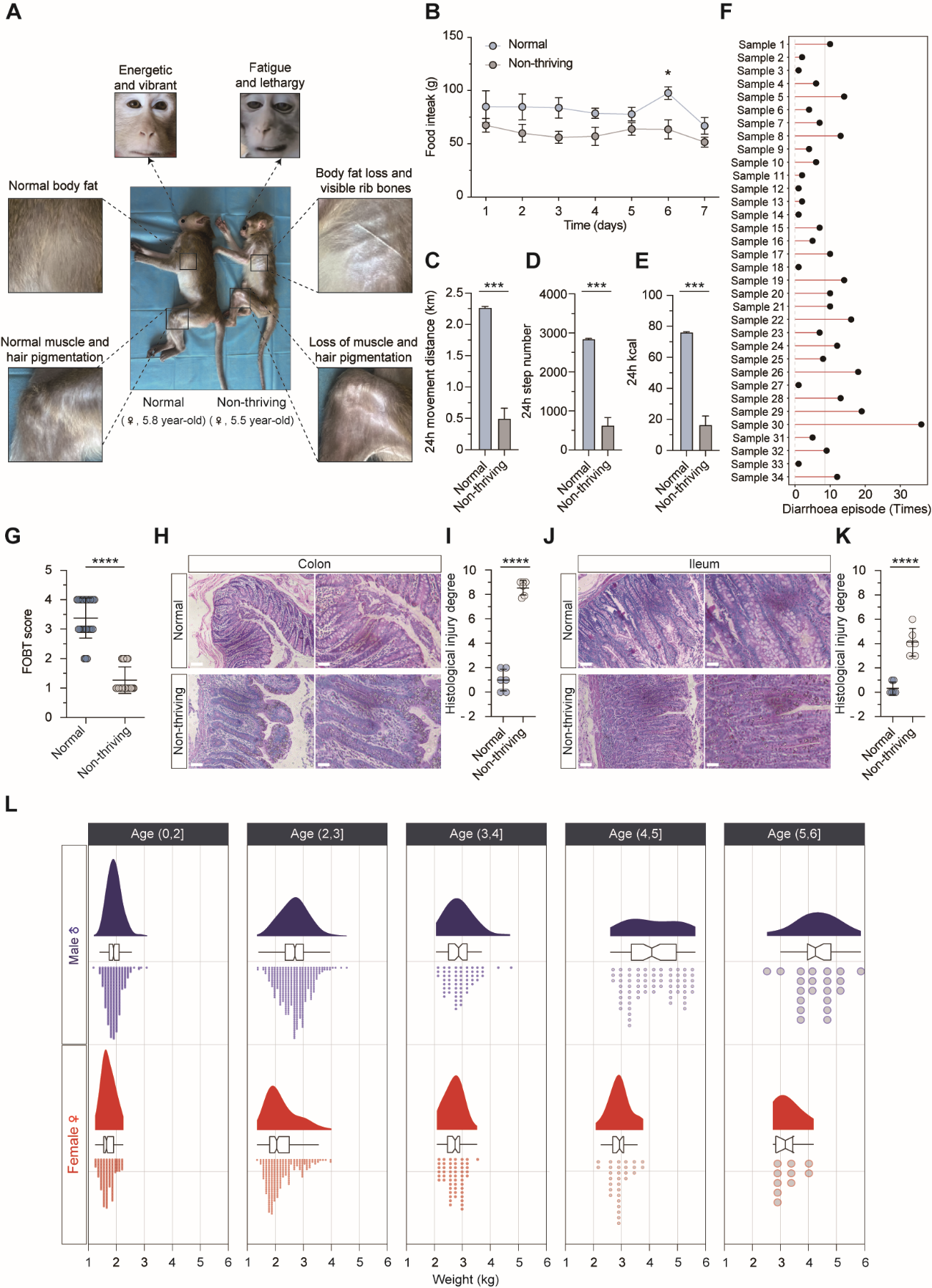
Figure. S1. Bodily characteristics of NHPs of different BCS scores**.

**(A)** Representative photographs of female NHPs of similar age with different BCS scores showing obvious differences in body fat and muscle mass, hair color pigmentation, dry skin and lesions. **(B)** Total 7-day food intake was general decrease in total food intake among the non-thriving NHPs relative to the normal counterparts. Movement status was counted using a portable pedometer fixed to the back of each monkey.Bar plot showing movement distance **(C)**, step number **(D)** and body energy consumption kcal **(E)** in 24 hours of normal and non-thriving NHPs. **(F)** Incidence of diarrhea episodes among non-thriving NHPs. **(G)** Fecal occult blood testing (FOBT) score showing significant intestinal bleeding in non-thriving NHPs compared to healthy looking counterparts. Postmortem HE staining showing crypt abscesses and basal plasma cell infiltration in the **(H,I)** colon and **(J,K)** ileum of non-thriving NHPs. Scale bar: Left 100 µm; right: 50 µm. **(L)** Age and weight distribution of the reference population of monkeys: Males (n = 857); Females (n = 530). Comparative analysis was done using independent student t-test. *: *P* < 0.05; **: *P* < 0.01; ***: *P* < 0.001, (n = 60: N-NHP = 20 and U-NHP = 40). NHP: Non-human primate; N-NHP: Normal non-human primate; U-NHP: Undernourished non-human primate.


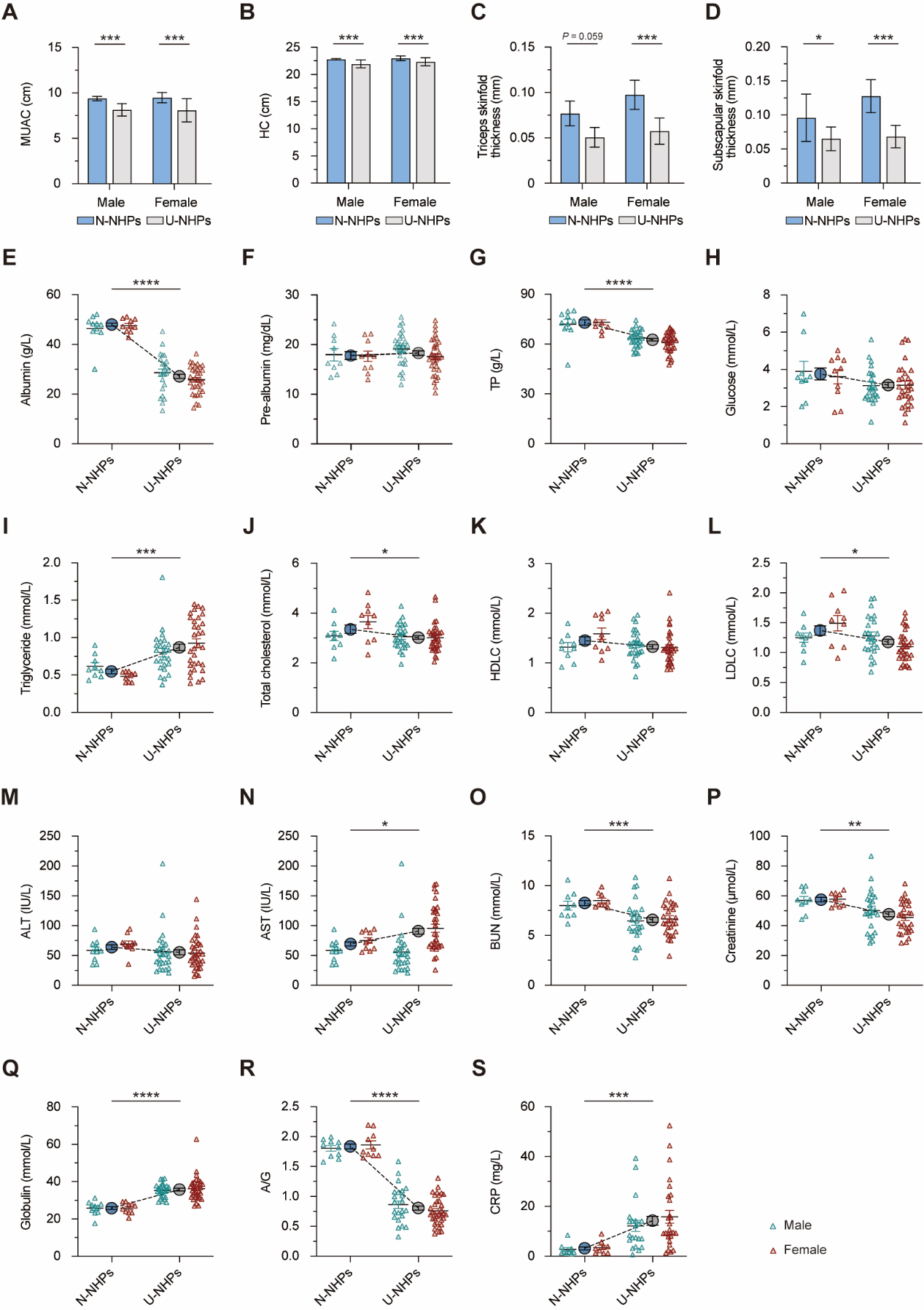


**Figure. S2. Biochemical profile of NHPs by gender**.

**(A-F)** Differences in MUAC **(A),** HC **(B)**, subscapular skinfold thickness **(C)** and triceps skinfold thickness **(D)** between the normal and non-thriving NHPs in the study cohort. Differences in levels of Albumin **(E)** Pre-albumin **(F)**, TP **(G),** Glucose **(H)**, TG **(I),** TC **(J)**, ALT **(K)**, AST **(L)**, HDLC **(M)**, LDLC **(N)**, BUN **(O)**, Creatinine **(P)**, Globulin **(Q)**, A/G **(R)** and CRP **(S)** according to gender between the N-NHP and U-NHPs. Statistical analysis was done using independent sample *t-test*. *: *P* < 0.05; **: *P* < 0.01; ***: *P* < 0.001; N-NHPs, n = 20 (10 males and 10 females); U-NHPs, n = 60 (24 males and 36 females). NHP: Non-human primate; N-NHP: Normal non-human primate; U-NHP: Undernourished non-human primate. TP: Total protein; TG: Triglycerides; TC: Total cholesterol; ALT: Alanine aminotransferase; AST: Aspartate transferase; HDLC: High density lipoprotein cholesterol; LDLC: Low density lipoprotein cholesterol; BUN: Blood urea nitrogen; A/G: Albumin/globulin ratio; CRP: C-reactive protein.

**
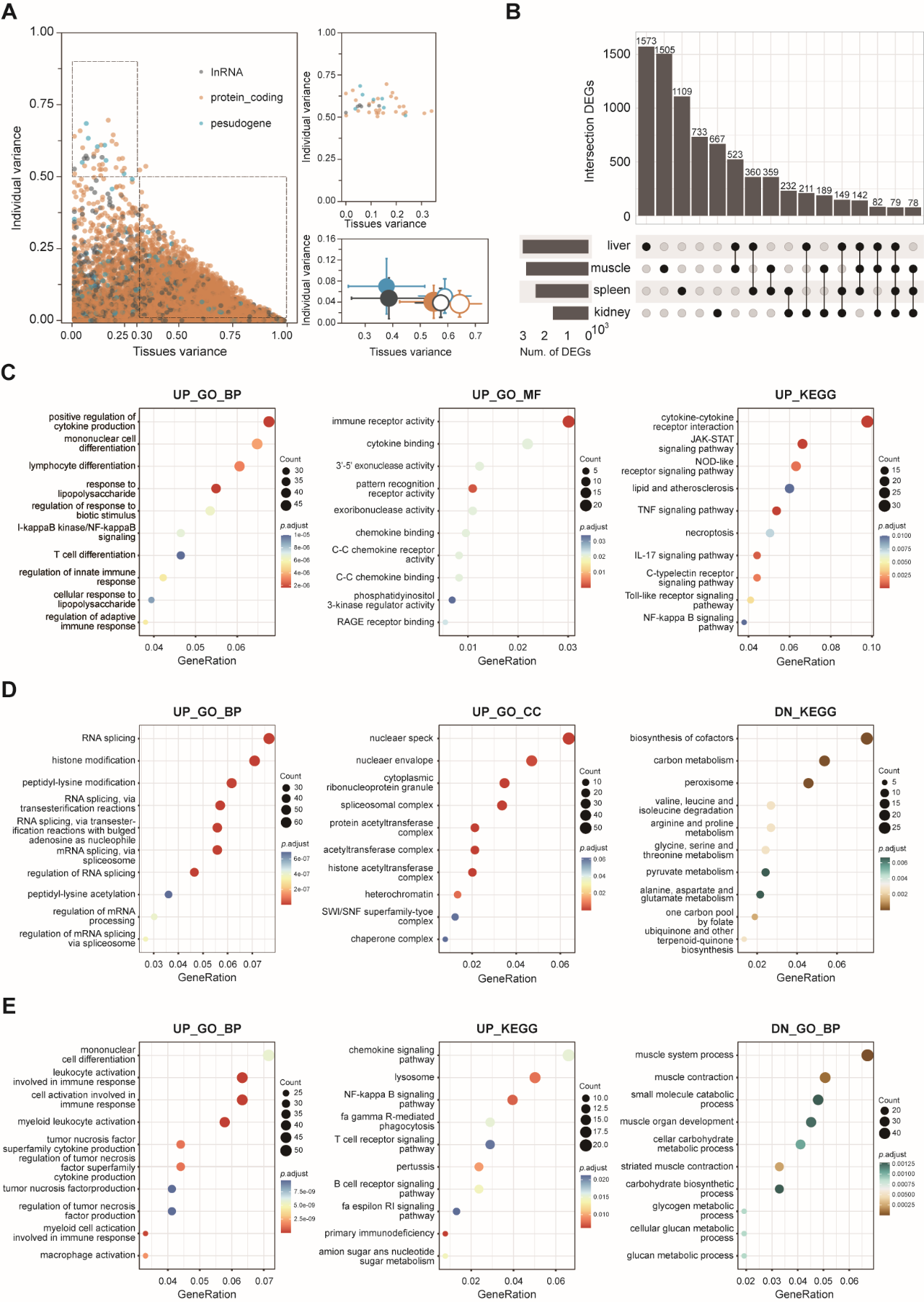
Figure. S3. DEGs are associated with dysregulation of metabolic pathways in U-NHPs**.

**(A)** Gene expression across tissues and individuals. **Left**: Contribution of individual variance and/or tissue variance to gene expression variation of lncRNAs, PCGs and pseudogenes. **Top right**: Genes with high individual variation and low tissue variation. **Bottom right**: Mean ± SD over all genes (filled circles) and over genes with similar expression levels in the PCGs and lncRNAs (unfilled circles). Circle size is proportional to the sum of individual and tissue variation and segment length corresponds to 0.5 SD. **(B)** Differentially expressed genes in the liver, muscle, kidney and spleen between N-NHPs and U-NHPs. **(C-E)** Gene ontology analysis of metabolic pathways associated with differentially expressed genes in the: spleen **(C)**, kidney **(D)** and muscle **(E)**. n = 3 for each group). Statistical analysis was done using independent sample *t-test*. *: *P* < 0.05; **: *P* < 0.01; ***: *P* < 0.001. N-NHP: Normal non-human primate; U-NHP: Undernourished non-human primate; UP: Up regulated; DN: Down regulated; GO: Gene ontology; BP: Biological process; MF: Molecular function; CC: Cellular component.


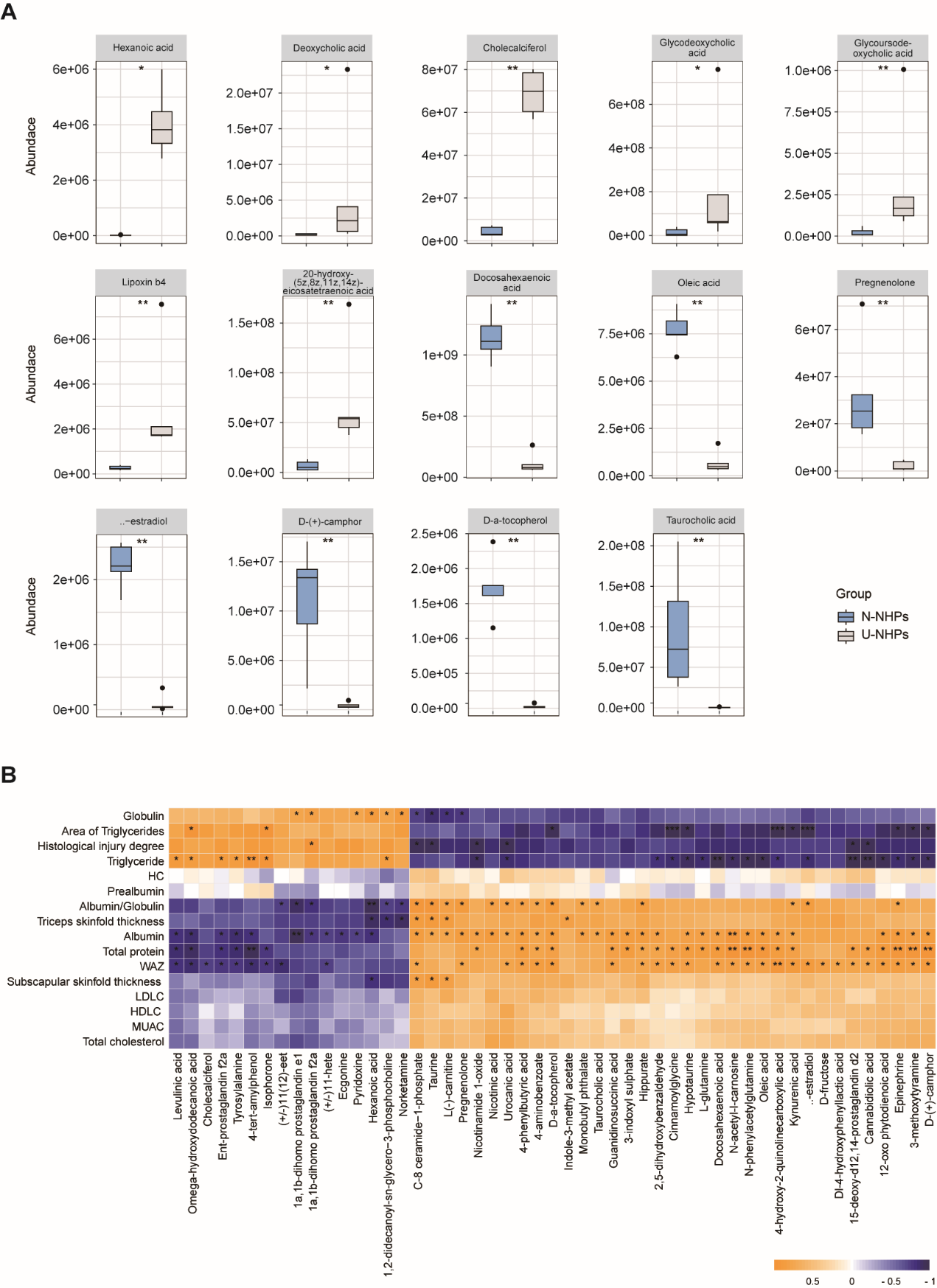


**Figure. S4. Metabolomic analysis reveal differentially abundant liver metabolites U-NHPs.**

**(A)** Comparative abundance of selected metabolites in the liver of N-NHPs and U-NHPs. **(B)** Correlation analysis showing associations between anthropometric measurements, serum metabolites and liver metabolites.

**
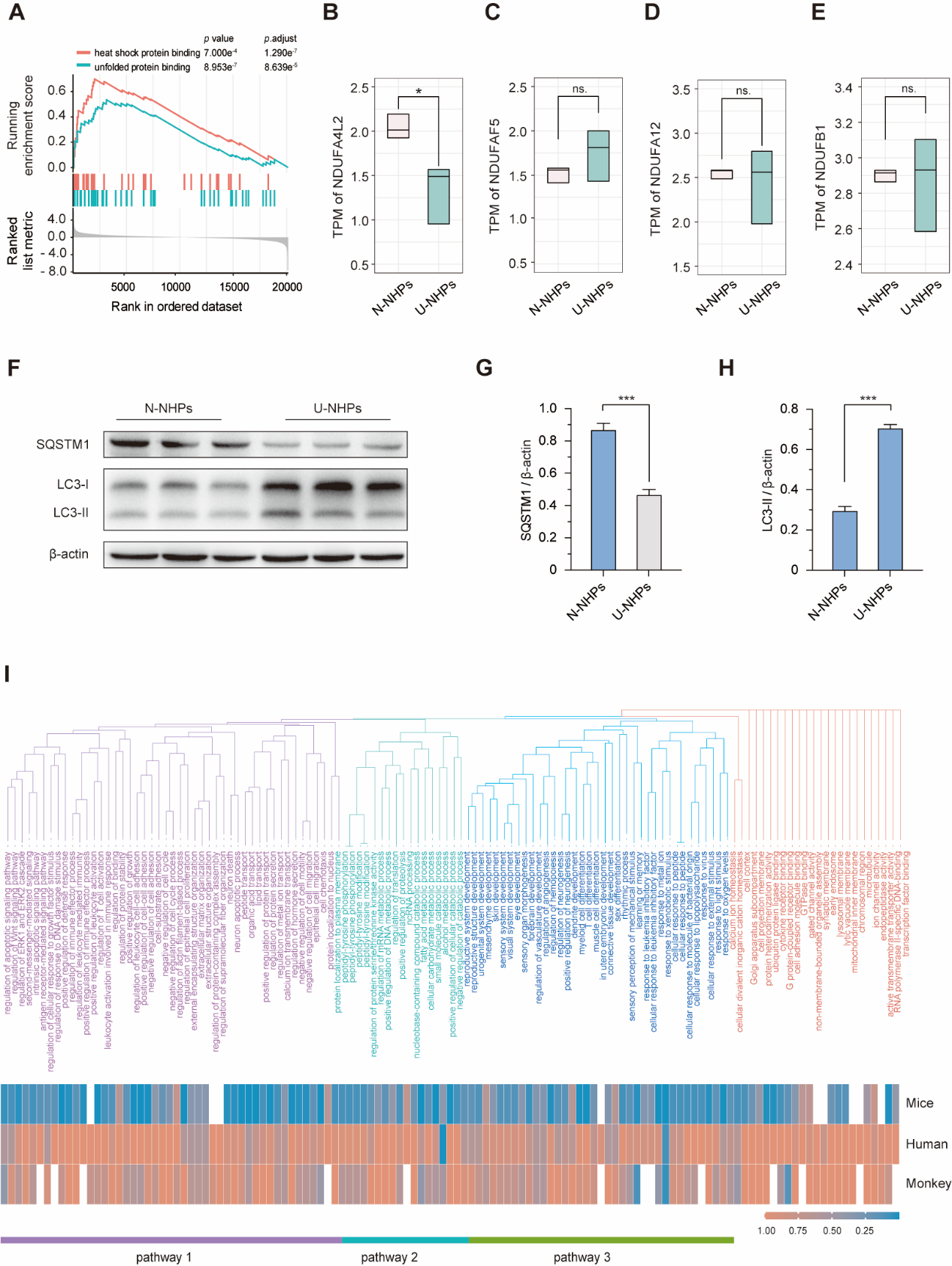
Figure. S5. Mitochondrial dysfunction is implicated in undernutrition-associated hepatic steatosis in U-NHPs**.

**(A)** GSEA plot of differentially expressed genes (DEGs)-associated enriched metabolic pathways in the liver of U-NHPs. **(B)** Relative expression level of OXPHOS complex IV-associated gene, NDUFA4L2. **(C-E)** Relative expression level of OXPHOS complex I-associated genes, NDUFAF5 **(C)**, NDUFA12 **(D)** and NDUFB1 **(E)**. (**F)** Western Blot analysis of SQSTM1 (p62) and LC3-II. **(G)** Quantification of the relative protein levels of p62 between N-NHPs and U-NHPs. **(H)** Quantification of the relative protein levels of LC3-II between N-NHPs and U-NHPs. (**I)** Heatmap showing specie differences in regulations of metabolic pathways under stress condition associated with lipid metabolism between rodents, NHPs and humans. Statistical analysis was done using independent sample *t-test*. *: *P* < 0.05; **: *P* < 0.01; ***: *P* < 0.001. N-NHP: Normal non-human primate; U-NHP: Undernourished non-human primate.

**
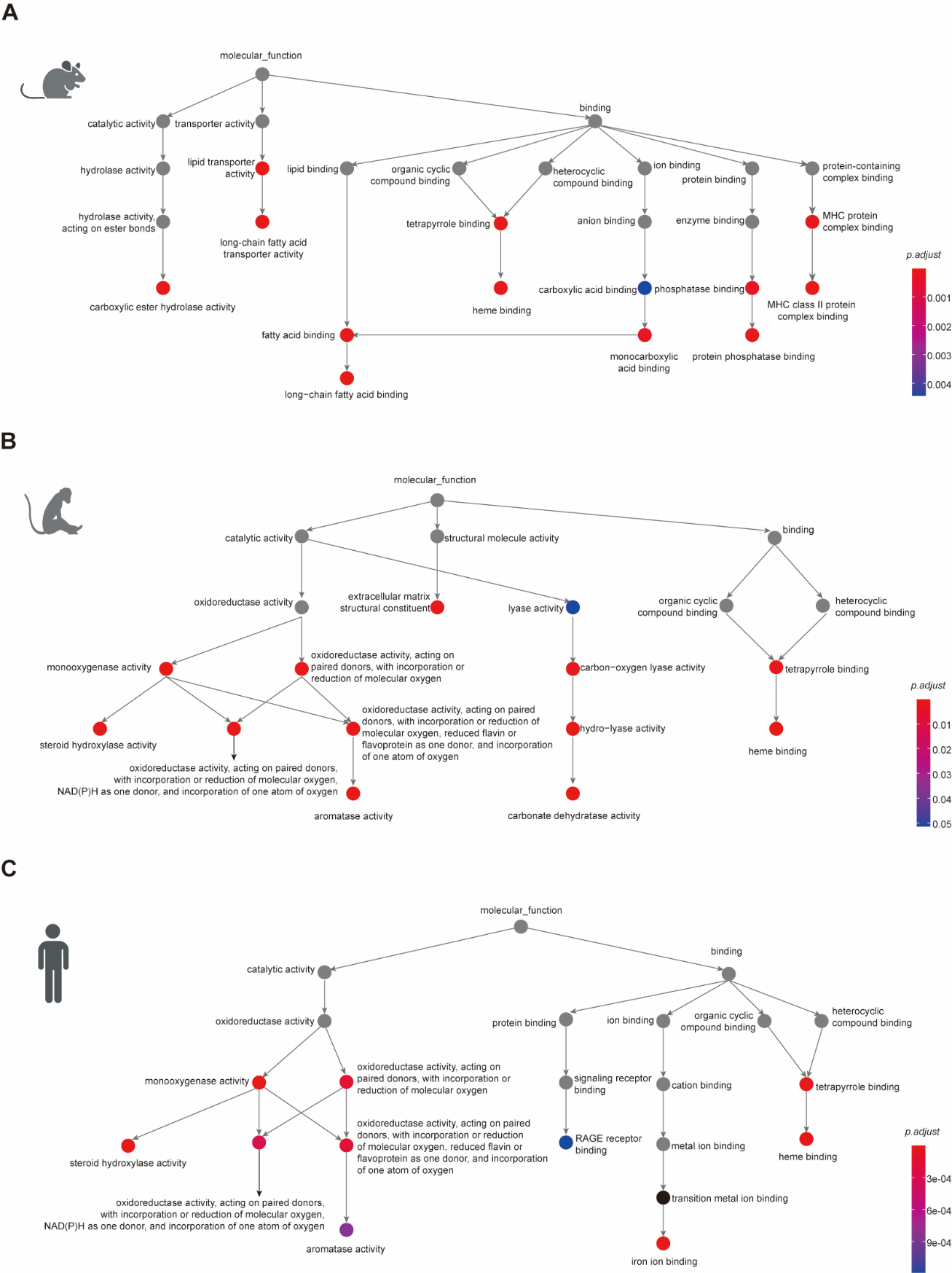
Figure. S6. Significant variations in fatty acid metabolic pathways across different species under fatty liver conditions.**

**(A)** Fatty acid metabolism pathways under fatty liver conditions in rodents. **(B)** Fatty acid metabolism pathways under fatty liver conditions in NHPs. **(C)** Fatty acid metabolism pathways under fatty liver conditions in humans.

**
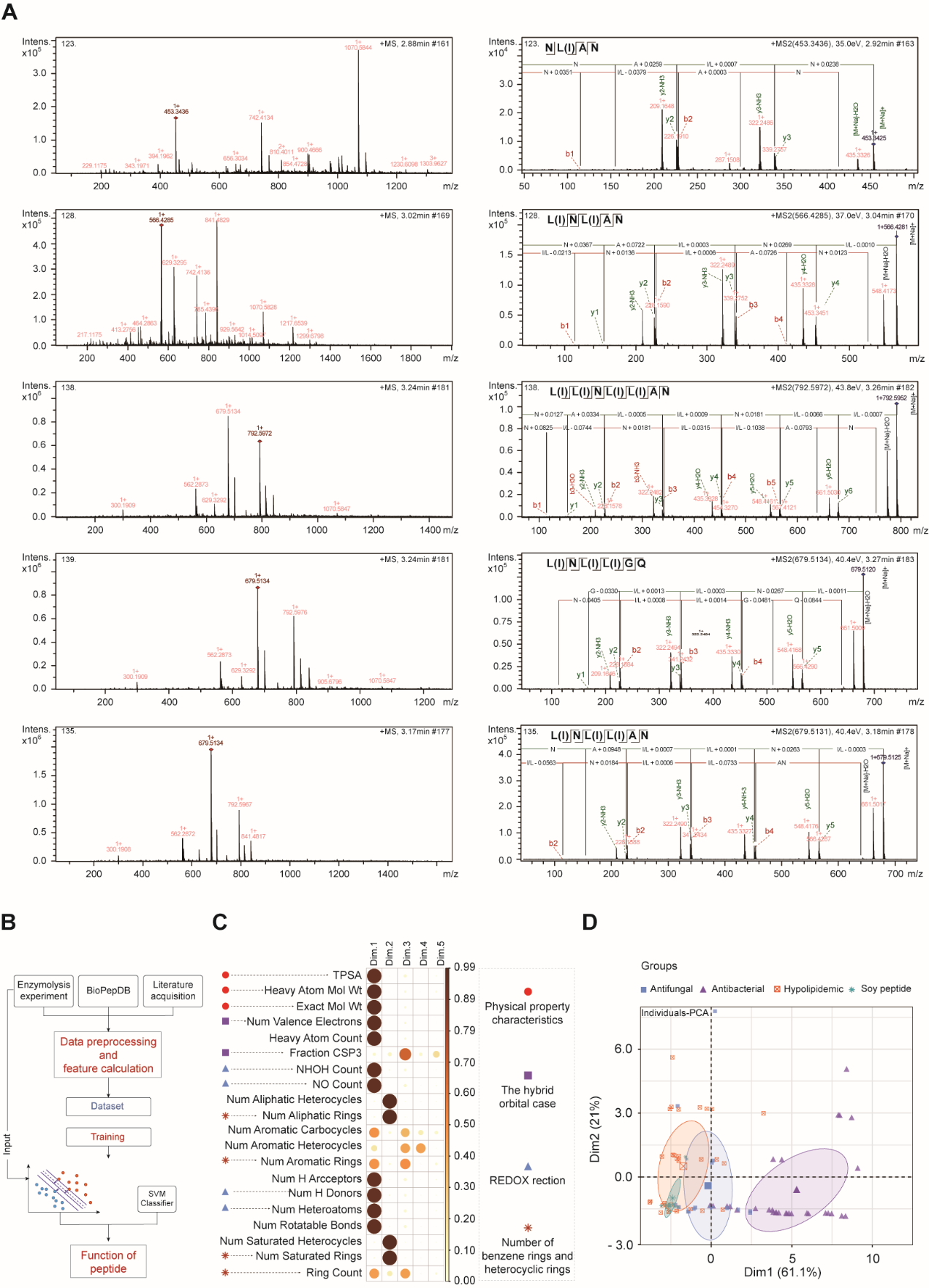
Figure. S7. Elucidation of hypolipidemic bioactive soypeptides**.

**(A)** **Representative** LC-MS/MS chromatographs of soypeptides showing enriched branched chain amino acids. **(B)** **Schematic flow** for the acquisition and functional prediction of the properties of bioactive peptides based on chemical molecular descriptors. **(C)** Comparative enriched chemical descriptors between different bio functional peptides. **(D)** PCA analysis showing the clustering together of Soypeptides and hypolipidemic peptides.


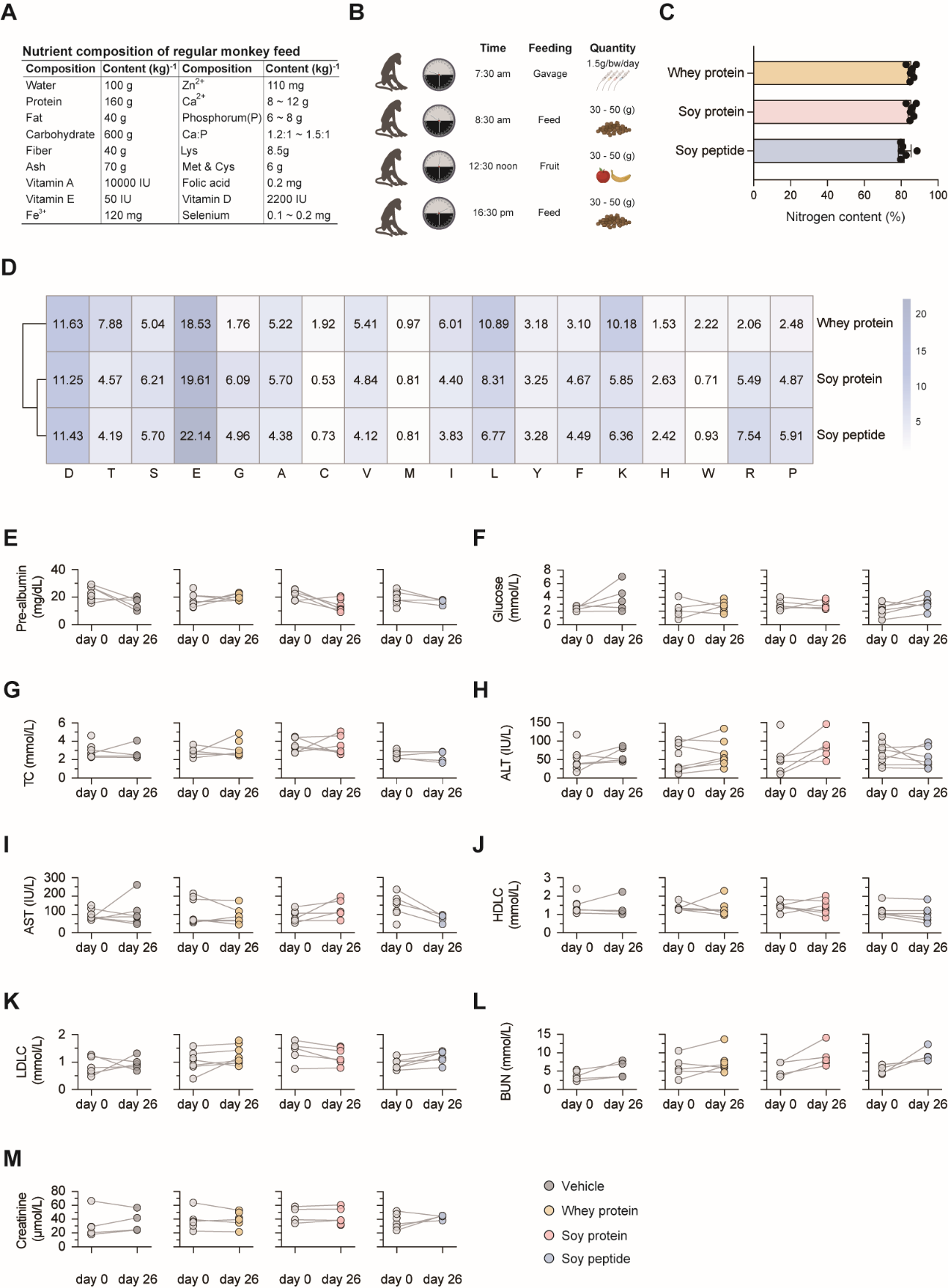


**Figure. S8. Biochemical characteristics of soypeptides and effect on serum biochemical profile in U-NHPs**.

**(A)** Nutrient composition of regular monkey feed. **(B)** Feeding schedule of NHPs at the experimental phase. **(C)** Total nitrogen content of whey protein, soyprotein and soypeptide supplements. **(D)** Percent amino acid composition of whey protein, soyprotein and soypeptide supplements. **(E-M)** Changes in levels of Pre-albumin **(E)** Glucose **(F**), TC **(G)**, ALT **(H)**, AST **(I)**, HDLC **(J)**, LDLC **(K)**, BUN **(L)**, Creatinine **(M)** in (Left to right) the vehicle-treated, whey protein-treated, soyprotein-treated and soypeptide-treated U-NHPs. Within group statistical analysis was done using paired sample t-test. *: *P* < 0.05; (n = 4 - 8). U-NHPs: Undernourished non-human primates; GSEA: Gene set enrichment analysis; GPC: Gel permeation chromatography; FTIR: Fourier transform infrared.


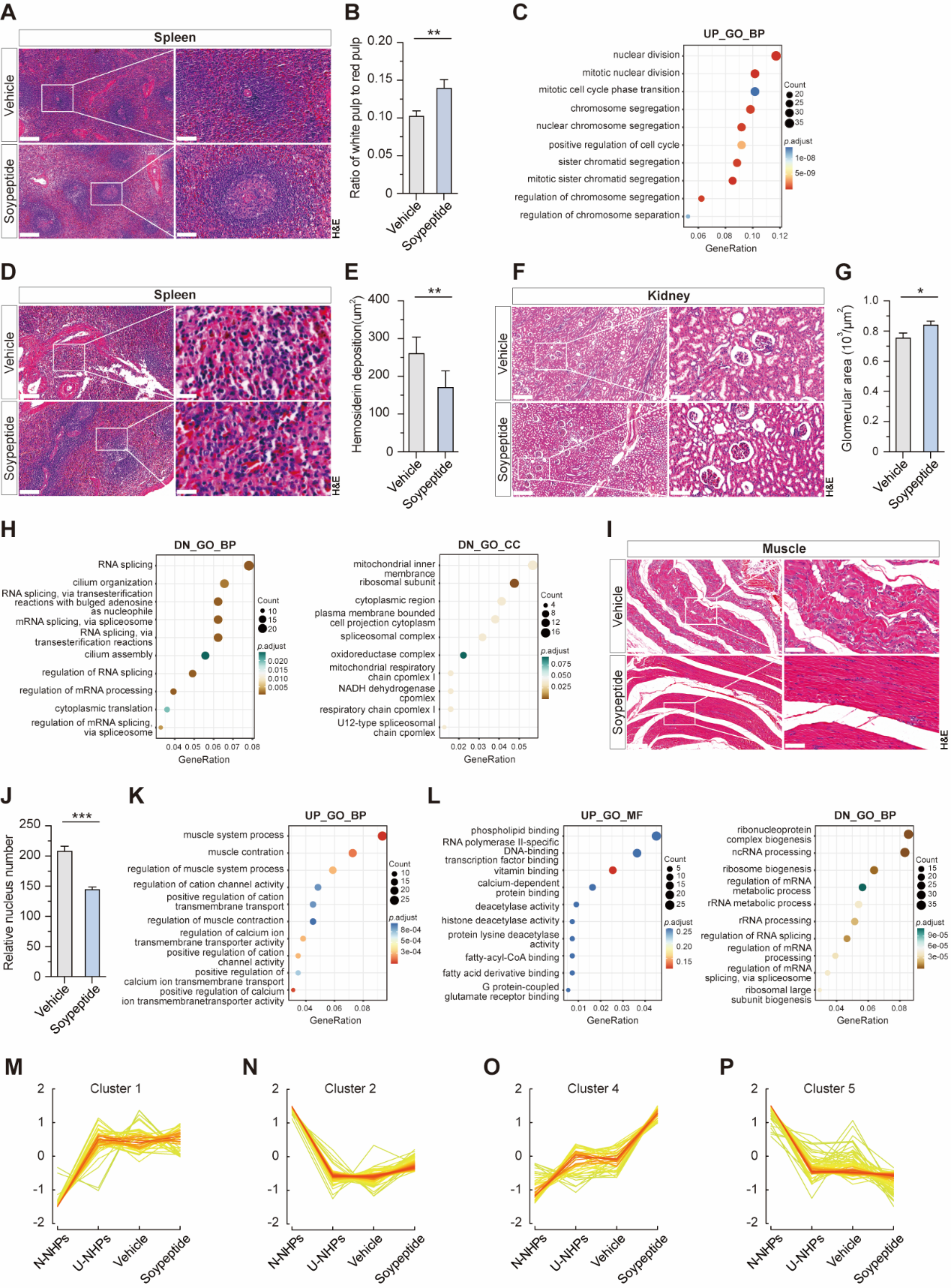


**Figure. S9.** **Soypeptide treatment rescues pathological injury in U-NHPs**.

**(A)** H&E staining of spleen sections showing recovery of white pulp and expansion of red pulp. Scale bar: 200 µm (left panel) and 50 µm (right panel). **(B)** Quantification of the relative ratio of white pulp to red pulp. **(C)** KEGG analysis of metabolic pathways associated with differentially expressed genes in the spleen. **(D)** H&E staining of spleen sections showing reduced hemosiderin deposition. Scale bar: 200 µm (left panel) and 50 µm (right panel). **(E)** Quantification of relative hemosiderin deposition. **(F)** H&E staining of kidney sections showing improved glomerular area in soypeptide-treated U-NHPs. Scale bar: 200 µm (left panel) and 50 µm (right panel). **(G)** Quantification of relative glomerular area. **(H)** KEGG analysis of metabolic pathways associated with differentially expressed genes in the kidney. **(I)** H&E staining of muscle sections showing reduced number of nuclei and improved muscle fiber architecture. Scale bar: 200 µm (left panel) and 50 µm (right panel). **(J)** Quantification of relative number of nuclei in muscle. **(K)** KEGG analysis of metabolic pathways associated with differentially expressed genes in the muscle. **(L)** KEGG analysis of metabolic pathways associated with differentially expressed genes in the liver. **(M-P)** Distinct clusters of liver metabolites identified using fuzzy c-means algorithm. n = 6 for each group. Statistical analysis was determined using ANOVA with post hoc Dunnett’s test. *: *P* < 0.05; **: *P* < 0.01; ***: *P* < 0.001. U-NHPs: Undernourished non-human primates; SFA: Saturated fatty acids; UFA: Unsaturated fatty acids; PUFA: Polyunsaturated fatty acids; MUFA: Monounsaturated fatty acid.
