# Supplementary material for: Soy Peptide Supplementation Mitigates Undernutrition through Reprogramming Hepatic Metabolism in a Novel Undernourished Non‐Human Primate Model
Source: Adv Sci (Weinh). 2024 May 30;11(29):2306890. doi: 10.1002/advs.202306890 (PMC11304262; doi:10.1002/advs.202306890)
Supplement: Supplementary file 3 — Supporting Information Appendix 1 [file ADVS-11-2306890-s003.pdf]

ADVANCED SCIENCE
Open Access

Supporting Information

for *Adv. Sci.*, DOI 10.1002/advs.202306890

Soy Peptide Supplementation Mitigates Undernutrition through Reprogramming Hepatic Metabolism in a Novel Undernourished Non-Human Primate Model

*Zhenzhen Xu, William Kwame Amakye, Zhengyu Ren, Yongzhao Xu, Wei Liu, Congcong Gong, Chiwai Wong, Li Gao, Zikuan Zhao, Min Wang, Tao Yan, Zhiming Ye, Jun Zhong, Chuanli Hou, Miao Zhao, Can Qiu, Jieqiong Tan, Xin Xu, Guoyan Liu, Maojin Yao and Jiaoyan Ren**

动物实验申请表

1. 概况

项目名称/编号	基于食蟹猴模式动物研究蛋白及肽类物质的营养功效与机制/ HZ2020074		
项目负责人	刘炜	部门	动物实验中心
电话	15977778772	邮箱	wei_liu@huazhengd.com
（√）初次提交		（ ）修改提交	
实验人员（包括所有涉及动物操作人员信息）			
姓名	部门	电话	邮箱
刘炜	动物实验中心	15977778772	wei_liu@huazhengd.com
苏科龙	动物实验中心	13660645085	kelong_su@huazhengd.com
杨波	动物实验中心	13457084890	bo_yang@huazhengd.com
许良知	动物实验中心	13802423579	liangzhi_xu@huazhengd.com
罗发高	动物实验中心	13700676352	fagao_luo@huazhengd.com

从化市华珍

2. 动物信息

物种/种系：食蟹猴

动物来源：从化市华珍动物养殖场（普通合伙）

动物数量：24 只

动物性别：18 只雌性，6 只雄性

年龄：2-8 岁

体重：1-1.5kg

预计实验时间：28 天

实验期间动物饲养方式 ☑ 单笼 （规格： cm ）

☐ 大栏 （规格： m）

实验期间水/饲料：

早餐：颗粒饲料，50g，约 09:00 喂食

中餐：水果，100g，约 12:00 喂食

晚餐：颗粒饲料，100g，约 16:00 喂食

3.实验目的

用简单易懂的文字简要说明实验目的及在维护人类/动物健康、增加知识或社会利益的重要性：
本实验针对食蟹猴肠道炎症(IBD)、肌肉萎缩、免疫功能损伤等症状，研究蛋白及肽类营养物质经灌胃后对上述食蟹猴机体营养状况、减少死亡率、改善肠道微生物环境、改善肠道炎症、调节肠道免疫等指标的有效性。本研究对于提高理解食物营养与功效具有重要意义，同时可以改善非人灵长类动物福利，同时提高非人灵长类实验动物养殖企业的效益具有重要意义。

4. 动物使用理由

（1）说明动物使用的理由。[包括为何不能使用非动物模型和低等动物模型的原因]

由于食物营养研究的复杂性，目前研究表明食源性功能营养因子对机体多种代谢紊乱及免疫功能降低具有较好的改善作用，然而大部分研究是基于啮齿类动物，而啮齿类动物因为近交系、夜行动物、具有食粪特性等，生化习性与人类相差甚远。此外，食品营养学研究中的一些生物学过程和机理难以通过体外模拟实现，因此选用食蟹猴为该实验的最佳动物模型。

（2）说明动物使用数量的合理性。[动物数量应限为统计学得到有效结论所需最少数量]

本实验所用营养不良食蟹猴均为自发性模型，与诱导性动物模型相比，其年龄、性别、发病情况均具有一定的差异，从而导致动物均一性较差，因此每组数量选择 8 只以得到具有统计学意义的数据。

5.实验设计和动物操作描述

（1）动物操作表 [请标明在本实验中要执行的动物操作]

[√]	注射或接种	[√]	血液/组织采集	[ ]	限制水及食物摄入
[ ]	行为改变	[√]	特别食物或液体治疗	[ ]	诱导产生的不适，中毒或疾病
[ ]	辐射	[ ]	动物保定用具使用	[ ]	较大存活手术
[ ]	小手术	[ ]	不可复原的手术操作	[ ]	繁殖
[√]	化学保定或麻醉	[ ]	其它：		

（2）简要描述实验设计并阐明所有动物操作 [让伦理委员会理解每只动物从进入到结束的整个实验过程]

- 实验过程中麻醉、止痛及镇静等特殊药物的使用

组别	特殊药物	给药剂量 (mg/kg)	给药容量 (ml/kg)	动物数量	给药途径	使用目的
所有 3 个组	盐酸氯胺酮	10	0.2	24 只	肌肉注射	诱导麻醉
所有 3 个组	戊巴比妥钠	100	3.0	24 只	静脉注射	安乐死

- 实验给药或接种方案 [化合物或生物制剂等]

组别	供试药物	给药剂量 (g/只)	给药容量 (ml/只)	动物数量	给药途径	药物溶剂
实验组 A	A	9	20	8	灌胃	纯水
实验组 B	B	9	20	8	灌胃	纯水
空白对照	无	0	20	8	灌胃	纯水

- 采血 [采血量、次数、部位及方法]

采血量：4ml/次；

次数：2 次；

部位及方法：四肢静脉采血。

- 动物预期要经受的不适感和应对措施 [例如疼痛或压迫等]

1. 抓取：动物抓取过程中会产生一定的应激，主要通过尽可能轻柔的动作，零食奖励等措施；
2. 采血：进针时会产生一定的疼痛和应激，主要采取良好的保定、术后给予零食奖励的措施；
3. 灌胃：插管和灌胃时会产生一定的应激与不适感，主要采取良好的保定、术后给予零食奖励的措施；
4. 安乐死：实验终点时需要安乐死部分动物采集标本，通过注射过量的麻醉剂来充分减少疼痛和恐惧。

- 其它应激源以及控制和减小动物痛苦的方法 [例如，限制水/食物摄入，伤害性刺激，环境紧张。如果实验的 USDA 疼痛分类为 E 类，请列出减轻疼痛和痛苦的非药物方法]

无

- 实验终点标准 [以体重增加或减少百分比，无能力吃或喝，行为异常，临床症状或中毒征兆等为实验终点，必须详细说明生物制剂，感染因子，辐射或危害性化学物质等在何时能引起显著的症状或致命。以死亡作为实验终点时必须有科学性评估]

1. 实验结束：到达实验结束日期 Day 28，猴子进行安乐死
2. 中途退出实验：动物在实验过程中如果出现不适合继续参与实验的状态时，该动物退出实验。

- 兽医护理计划 [一旦有动物疾病发生时的行动计划]

1. 每日兽医进行 2 次临床观察，及时发现异常症状；
2. 如发现有动物处于伤病状态及时按照 SOP 进行相应的处置；
3. 如发现有不适合继续进行实验的动物，及时建议项目负责人将改动物退出实验。

● 简要实验过程描述 [从动物进入实验室到实验结束后动物离开的整个过程]

适应期：实验前 7 天，动物由大栏移至单栏饲养，2 只/笼，适应 7d；

实验中：Day0 称体重、测体长、肛拭子收集粪便、静脉采血 3-4ml，分离 PBMC 及血清；

Day1-Day28 每日灌胃，每周称量体重；

Day28 测量皮褶厚度、臂围，无菌棉签肛拭子收集粪便，部分-80℃保存部分测量隐血情况，手臂静脉采血 3-4ml，分离 PBMC 及血清；

实验结束后：进行解剖，收集脏器，按实验需求对组织进行相应保存。

6.外科手术

如需手术，完成下面内容：

1. 简要描述手术方案 [包括术前操作（例如禁食、止痛药使用等）术中监护和护理及无菌方法等]

   不适用

2. 谁是手术操作者，资历经验如何？

   不适用

3. 在何地执行手术和术后护理？

   不适用

4. 如果是生存手术，描述术后护理要点，观察频率，指明责任人 [包括工作时间，下班时间，周末和假期期间的护理以及术后并发症的监测与护理]

   不适用

5. 如果是非生存手术，描述如何制定实验终点以及如何确认动物死亡。

   不适用

6. 手术中是否使用麻醉剂？如果是，请描述如何保持通风换气以及如何评估疼痛。

   不适用

7. 如果是重大生存手术，之前是否执行过预实验？[重大生存手术是指穿透或暴露身体内腔，或者产生实质性的身体损伤，或者损伤生理功能的手术，例如：剖腹手术，开胸手术，开颅手术，关节置换，截肢等]

   如果是，请说明：

   不适用

8. 是否存在多次或者多种重大生存手术执行于单只动物身上？如果是，请说明：

   不适用

7.疼痛或应激分类

☐ **B类：**为了教学、检测、研究或手术等目的而对动物饲养，但动物尚未被使用，所引起的疼痛或痛苦。

- 动物被饲养在尺寸符合规定的饲养笼内，且按照相关法规来对待动物。饲养种群包括父母代和后代。
- 新引进的动物被饲养在合理的饲养笼内，且按照相关法规来对待动物。
- 动物饲养在合适的且能够被观察的圈养条件下。

☑ **C类：**在教学、研究、实验或检测过程中，执行无疼痛或无痛苦的操作。或者执行需要使用缓解疼痛的药物来减轻动物的疼痛或痛苦的操作。

- 动物操作由受过培训的人正确执行，包括注射给药，口服给药，从外周静脉或其他标准兽医方法进行的血液采集，做放射图像，非刺激物质的注射给药等。
- 执行正确的安乐死方法。
- 人为约束动物，例如短时间的猴椅约束（猴椅约束时间在5小时以内）。

☐ **D类：**在教学、研究、实验或检测过程中可引起动物不适和疼痛，需要使用恰当的麻醉药、止痛药或镇静药来减轻动物的疼痛和不适。

- 由受过培训的人用标准兽医方法进行外科手术操作。包括取活检，生殖腺切除，血管暴露，慢性导管植入，开腹手术或腹腔镜检查。
- 通过危险途径采集血液，例如心脏内和眶周采血。
- 药物、化合物，毒物或微生物给药后引起的疼痛和不适，但通过使用止痛药减轻这些疼痛和不适。

☐ **E类：**在教学、研究、实验或检测过程中引起的动物疼痛，因麻醉药，止痛药或镇静药的使用会反方向影响到操作、教学解释或研究结果，因而不能使用药物来缓解疼痛或者使用药物后没有明显止痛效果。

- 实验操作引起的疼痛无法用止痛药减轻，例如毒理实验、微生物毒性测试、放射病以及紧张，休克及疼痛研究。
- 体腔手术，骨科手术，牙组织或其它硬、软组织损伤等手术和手术后遗症引起疼的疼痛或痛苦，且不能缓解。
- 通过电击等方法来制约、训练动物行为为导致的疼痛。
- 在未进行实验操作的情况下对非人灵长类动物进行长时期猴椅约束（约束时间在24小时以上）。

8. 实验结束后动物的安乐死方法及相关处置

即便实验结束时不安乐死动物，也需准备一个应急方案来应对意外伤害或不适。如果过量麻醉是应急方案，列出药品名称，剂量，给药途径。安乐死必须遵守正确的方法。
所有执行安乐死的人员必须经过培训并清楚了解确定死亡的方法。执行安乐死的培训记录必须有案可查。

品种/系	方法	药物	剂量 (mg/kg, % or 浓度)	途径
食蟹猴	过量麻醉法	戊巴比妥钠	100mg/kg	静脉注射
两名以上兽医严格按 SOP 执行安乐死并共同确认动物死亡，动物尸体进行焚烧火化。				

9. 本实验的特殊关注点或要求

列出任何特殊的饲养条件、设备、动物护理（例如，特殊笼、水、食物、废物处理、环境改善等）：

不适用

动物实验伦理委员会审查意见表

申请表编号：HZ2020074	项目负责人姓名：刘炜
项目名称/编号：基于食蟹猴模式动物研究蛋白及肽类物质的营养功效与机制/ HZ2020074	

（一）审查项目			综合审查意见
项次	实验设计及动物实验内容	结果	全体伦理委员会成员开会讨论后，一致通过该审查，同意该实验的进行
1	该实验没有重复以前的实验内容	是	
2	该实验不能用非动物模型模拟动物实验	是	
3	所选的动物品种（系）最适合于该实验	是	
4	所使用的动物数量是该实验取得理想实验结果的最少数量	是	
5	该实验设计合理，使用方法得当	是	
6	实验设计体现了善待动物的原则	是	
7	动物实验人员具备动物实验资格	是	
8	使用危险性物质（如生物感染性物质、放射性物质、基因重组物等）于本实验，得到相关部门许可，防范措施比较充分	是	
9	该实验结束后动物处理方案符合规定	是	
10	该实验动物尸体、标本、废弃物的处理方案符合规定	是	

（二）审查结果

☑【通过】

☐【待修正后再审查】

☐【不通过】

理由：

1. 试验设计科学合理，具备良好的科学价值，有开展的必要性；
2. 试验机构具有相关资质和各项硬件条件，试验人员和兽医人员具备开展此试验的技术能力；
3. 试验过程中动物可能遭受的痛苦、刺激和压力有较为妥善的处置措施。

伦理委员会主任签名：黄志锋	2020 年 09 月 20 日
其他审查者签名：[illegible] [illegible] [illegible] [illegible]	2020 年 09 月 20 日
