# Supplementary material for: Soy Peptide Supplementation Mitigates Undernutrition through Reprogramming Hepatic Metabolism in a Novel Undernourished Non‐Human Primate Model
Source: Adv Sci (Weinh). 2024 May 30;11(29):2306890. doi: 10.1002/advs.202306890 (PMC11304262; doi:10.1002/advs.202306890)
Supplement: Supplementary file 4 — Supporting Information Appendix 2 [file ADVS-11-2306890-s002.pdf]

ADVANCED SCIENCE
Open Access

# Supporting Information

for *Adv. Sci.*, DOI 10.1002/advs.202306890

Soy Peptide Supplementation Mitigates Undernutrition through Reprogramming Hepatic Metabolism in a Novel Undernourished Non-Human Primate Model

*Zhenzhen Xu, William Kwame Amakye, Zhengyu Ren, Yongzhao Xu, Wei Liu, Congcong Gong, Chiwai Wong, Li Gao, Zikuan Zhao, Min Wang, Tao Yan, Zhiming Ye, Jun Zhong, Chuanli Hou, Miao Zhao, Can Qiu, Jieqiong Tan, Xin Xu, Guoyan Liu, Maojin Yao and Jiaoyan Ren**

# Ethical application for animal experiments

## 1.General Overview

| Project name/number | Nutritional efficacy and mechanisms of action of proteins and peptides supplementation in cynomolgus monkeys /HZ2020074 | | |
|---|---|---|---|
| Project leader | Wei Liu | Department | Experimental Animal Center |
| Phone | 15977778772 | Email | wei_liu@huazhengd.com |
| (√) Initial submission | | ( ) Revised submission | |
| Study Team (Include information on all individuals involved in handling animal) | | | |
| Name | Department | Phone | Email |
| Wei Liu | Experimental Animal Center | 15977778772 | wei_liu@huazhengd.com |
| Kelong Su | Experimental Animal Center | 13660645085 | kelong_su@huazhengd.com |
| Bo Yang | Experimental Animal Center | 13457084890 | bo_yang@huazhengd.com |
| Liangzhi Xu | Experimental Animal Center | 13802423579 | liangzhi_xu@huazhengd.com |
| Fagao Luo | Experimental Animal Center | 13700676352 | fagao_luo@huazhengd.com |
| | | | |

## 2.Information about animals

Species/germ line： *Macaca fascicularis*
Animal origin： Conghua Huazhen Animal Breeding Farm （Ordinary partner）
Animal number： 24
Sex of animals： 18 females, 6 males
Age： 2-8 years
Body weight： 1-1.5 kg
Estimated experimental duration： 28 day
Housing situation during the experimental period：
☐√ Single cage （Size： cm ）
☐ Large column （Size： m）
Water/feed during the experiment：

Breakfast: pelleted feed, 50g, fed at about 09:00

Lunch: fruit, 100g, fed at about 12:00

Dinner: Pellet feed, 100g, fed at about 16:00

## 3.Experimental objective

| |
|---|
| Provide a brief explanation of the purpose of the experiment and its importance in the maintenance of human/animal health, the advancement of knowledge or the benefit of society in simple and |

understandable language:

In this study, we aimed to investigate the effectiveness of gastric administration of protein and peptides on the nutritional status, mortality reduction, improvement in the intestinal microbial environment, improvement in intestinal inflammation, and regulation of intestinal immunity in inflammatory bowel disease (IBD), muscle atrophy, and immunocompromised cynomolgus monkeys. This study has important implications for improving the understanding food nutrition efficacy, improving the welfare of nonhuman primates, and improving the profitability of nonhuman primate laboratory animal breeding enterprises.

## 4. Rationale for Animal Use

(1) State the rationale for animal use. [includes reasons why non-animal models and lower animal models cannot be used]

Although food nutrition research is complex, current studies have shown that functional nutrition factors have a good effect in improving a variety of metabolic and immune function disorders. However, most of the studies are based on rodent models which are very different from humans in biochemistry due to inbreeding, circadian rhythm, and feeding practice characteristics. In addition, some biological processes and mechanisms in food and nutrition research are difficult to replicated *in vitro*, so the cynomolgus monkey is the best animal model for this study

(2) Explain the rational for the number of animals used. [The number of animals should be limited to the minimum number required for statistically valid conclusions]

All malnourished cynomolgus monkeys used in this experiment were spontaneous models, and compared with the induced animal models, there existed significant differences in incidence rate with regards gender, age and general morbidity an, which resulted in poor animal model diversity. Therefore, the 8 cynomolgus monkeys for each group was selected to obtain statistically significant data.

## 5.Experimental design and animal handling description

(1) Animal Handling Table [Please indicate the procedures to be performed on animals in this experiment]

| | | | | | |
|---|---|---|---|---|---|
| [√] | An injection or vaccination. | [√] | Blood/tissue collection | [ ] | Limit water and food intake |
| [ ] | Behavior change | [√] | Special food or fluid therapy | [ ] | To induce discomfort, intoxication, or illness |
| [ ] | Radiation | [ ] | Animals are [illegible] | [ ] | Major SURVIVAL surgery |
| [ ] | Minor operation | [ ] | Irreversible surgical procedures | [ ] | Breed |
| [√] | Chemical Baoding or anesthesia | [ ] | Others: | | |

(2) Briefly describe the experimental design and clarify all animal procedures [to allow the ethics committee to understand the entire experimental process for each animal from entry to completion]

- The use of special drugs such as anesthesia, analgesia and sedation during the experiment

| Group | Special drugs | dosage of administration (mg/kg) | Volume of drug administered (ml/kg) | Animal number | Administration route | Application aim |
|---|---|---|---|---|---|---|
| All 3 groups | Ketamine hydrochloride | 10 | 0.2 | 24 | Intramuscular injection | Induced anesthesia |
| All 3 groups | Pentobarbital sodium | 100 | 3.0 | 24 | Intravenous injection | Euthanasia |

- Drug administration or inoculation protocols [compounds or biological agents, etc.]

| Group | Test drugs | Dosage of administration (mg/kg) | Volume of drug administered (mL/animal) | Animal number | Administration route | Solvent for administration |
|---|---|---|---|---|---|---|
| Experimental group A | A | 9 | 20 | 8 | Gavage | Water |
| Experimental group B | B | 9 | 20 | 8 | Gavage | Water |
| Control group C | None | 0 | 20 | 8 | Gavage | Water |

- Blood collection [blood collection volume, frequency, location and method]

Blood collection volume: 4ml/ time;

Frequency: 2 times;

Location and method: Blood collection from the veins of the extremities.

- The expected discomfort to animal and the response (e.g. pain or compression, etc.)

  1. Grasping: There will be a certain stress in the process of grasping. To minimize this, movement will be done as gentle as possible, snack rewards and other measures;

  2. Blood collection: there will be a certain level of pain and stress when the needle is inserted. Guarantee good procedures and provide postoperative snack reward.

  3. Intragastric administration: certain stress and discomfort would occur during intragastric administration. Guarantee good administration procedure and provide snacks rewards after the operation.

  4. Euthanasia: At the end of the experiment, part of the animal needs to be euthanized for specimen collection, and the pain and fear will sufficiently be reduced by injecting an appropriate amount of anesthetic

- Other stressors and methods to control and reduce animal pain [e.g., water/food restriction, noxious stimuli, environmental stress. If the experiment uses USDA pain classification class E, list non-pharmacological methods to reduce pain and suffering]

  None

- End point criteria [percentage weight gain or loss, inability to eat or drink, abnormal behavior, clinical symptoms or signs of poisoning, etc., as the end point of the trial, must specify when biological agents, infectious agents, radiation or hazardous chemicals, etc., can cause significant symptoms

or be fatal.

1. End of experiment: At the end of the experimental Day 28, the monkeys will be euthanized

2. Withdrawal from the study: If an animal develops any condition making unsuitable to continue or participate in the experiment, the animal will be withdrawn from the study.

- Veterinary care plan [action plan in case of animal disease]

  1. Clinical observation will be carried out twice a day by veterinarians to detect abnormal symptoms in time.

  2. If any animal is found to injury, there will be timely handling according to the SOP;

  3. If there are any animals that are not suitable for continuing the experiment, veterinarian will provide timely advise to the project leader to withdraw the animal from the experiment.

- Brief description of the experimental process [the entire process from the animal entering the laboratory to the animal leaving after the end of the experiment]

  Adaptation period: 7 days before the experiment, the animals will be moved from the large pen to the single pen, 2 animals/cage, to allow for 7 days of adaptation.

  During the experiment:

  Day0: taking body weight, measured body length, anal swab for stool collection, venous blood collection of 3-4ml, separation of PBMC and serum.

  From Day1 to Day 28: Daily gavage, and the body weight measurement every week.

  On Day 28: skinfold thickness and arm circumference measurement. Fecal samples collection with sterile cotton swabs, and sample storage at -80℃ to measure occult blood.

  At the end of the experiment: dissection will be performed, organs excised, and tissues preserved according to the experimental requirements.

**6.Surgery**

If surgery is required, complete the following:

1. Briefly describe the surgical protocol [including preoperative procedures (e.g., fasting, painkiller use, etc.), intraoperative monitoring and care, and aseptic methods, etc.]

Not applicable

2. Who are the operators and what are their qualifications and experiences?

Not applicable

3. Where will the surgery and postoperative care be performed?

Not applicable

4. If it is a survival operation, describe the key points of postoperative care, observation frequency, and identify the responsible person [including working hours, off hours, weekend and holiday care and postoperative complications monitoring and care]

Not applicable

5. If a non-survival procedure is performed, describe how trial endpoints are formulated and how death of the animal is confirmed.

Not applicable

6. Is anesthetic used during the operation? How to evaluate the effect of anesthesia? How to maintain ventilation when using gas anesthesia?

Not applicable

7. If it is a major survival surgery, has a pilot study been performed? [Major survival surgery refers to surgery that penetrates or exposes internal cavities of the body, or causes substantial physical injury, or impairs physiological functions, such as: laparotomy, thoracotomy, craniotomy, joint replacement, amputation, etc.]

If yes, please state:

Not applicable

8. Were there multiple or major survival operations performed on a single animal? If yes, please state:

Not applicable

**7.Pain or stress classification**

☐ Class B: The keeping and breeding of animals for purposes such as teaching, testing, research, or surgery.

☐√ Class C: In the course of teaching, research, experimentation, or testing, a painless or painless operation is performed. Or perform procedures that require the use of pain-relieving drugs to relieve pain or suffering in the animal.

- Animal handling should be properly performed by a trained person and includes administration of drugs by injection, administration of drugs by mouth, blood collection from a peripheral vein or other standard veterinary methods, injection of non-irritating substances, etc.
- Perform correct euthanasia methods
- Manual restraint of animals, such as monkey chair restraint for short periods (monkey chair restraint duration < 5 hours)

☐ Class D: Cause discomfort and pain in animals during teaching, research, experiments or testing, and it is necessary to use appropriate anesthetics, painkillers or sedatives to reduce the pain and discomfort of animals.

- Surgical procedures performed by trained personnel using standard veterinary methods These include taking a biopsy, gonectomy, vascular exposure, chronic catheter implantation, open surgery or laparoscopy.
- Collect blood from risky routes, such as intracardiac and periorbital sampling.
- Pain and discomfort caused by administration of a drug, compound, toxicant, or microorganism, but reduced by the use of pain medication

☐ Class E: animal pain caused in the course of teaching, research, experiment or detection, because the use of anesthetics, analgesics or sedatives will adversely affect the operation, teaching interpretation or research results, so that drugs cannot be used to relieve pain or have no obvious analgesic effect after the use of drugs.

- Pain caused by experimental procedures that cannot be alleviated with analgesics, such as toxicology experiments, microbial toxicity testing, radiation sickness, and stress, shock, and pain studies.
- Painful pain or suffering caused by surgery and sequelae of surgery, such as cavity surgery, orthopedic surgery, dental tissue or other hard or soft tissue injury, without relief.
- Pain caused by conditioning and training animal behavior through methods such as electric shocks.

- Use of prolonged monkey chair restraint (> 24 hours of restraint) on nonhuman primates without experimental manipulation.

**8.Methods of euthanasia and related disposal of animals at the end of the experiment**

Even if the animals are not euthanized at the end of the experiment, a contingency plan should be prepared in the event of accidental injury or discomfort. If overdose is an emergency, list the drug name, dose, and route of administration. Euthanasia must follow the correct method.

All those performing euthanasia must be trained and clearly understand the methods used to determine death. Training in euthanasia must be documented.

| Breed/line | Method | Drug | Dose (mg/kg,% or concentration) | Approach |
|---|---|---|---|---|
| *Macaca fascicularis* | Overdose anesthesia method | Sodium pentobarbital dissociation | 100mg/kg | Intravenous injection |
| Two or more veterinarians will perform euthanasia strictly according to the SOP and jointly confirmed the death of the animal, and the animal carcass will be was burnt and cremated. | | | | |

**9.Special concerns or requirements of this experiment**

List any special feeding conditions, equipment, animal care (e.g. special cages, water, food, waste disposal, environmental improvement, etc.) :

Not Applicable

Review comment form of the Ethics Committee on Animal Experiments

| Application No.: HZ2020074 | Name of project leader: Wei Liu |
|---|---|
| To investigate the nutritional effects and mechanisms of proteins and peptides in cynomolgus monkeys /HZ2020074 | |

| (1) Items to review | | | Comprehensive review comments |
|---|---|---|---|
| Item | Experimental design and contents of animal experiments | Result | The review was unanimously approved by all ethics committee members after a meeting and discussion, and the experiment was approved |
| 1 | The experiment did not replicate the contents of previous experiments | Yes | |
| 2 | The experiment cannot be simulated with non-animal models for animal experiments | Yes | |
| 3 | The selected animal breed (line) was most suitable for the experiment | Yes | |
| 4 | The number of animals used was the minimum number for the experiment to achieve the desired experimental result | Yes | |
| 5 | The design of the experiment is reasonable and the method used is appropriate | Yes | |
| 6 | The experimental design embodies the principle of animal kindness | Yes | |
| 7 | Animal experiment personnel are qualified for animal experiments | Yes | |
| 8 | The use of hazardous substances (such as biological infectious substances, radioactive substances, genetic recombinant substances, etc.) in this experiment was approved by the relevant departments, and the preventive measures were adequate | Yes | |
| 9 | At the end of the experiment, the animal treatment protocol was in accordance with the regulations | Yes | |
| 10 | The disposal scheme of the experimental animal carcasses, specimens and wastes was in accordance with the regulations | Yes | |

☐√【 Pass 】
☐【 Review after correction 】
☐【 Fail 】

Reasons:
1. The experimental design was scientific and reasonable
It has good scientific value and the necessary for development.
2. The test institution has the relevant qualifications and hardware conditions, test personnel and veterinary personnel have the technical ability to carry out this test :3. The pain, stimulation and stress that animals may suffer during the experiment could be handled properly.

| | |
|---|---|
| Signature of the Director of the Ethics Committee | Data |
| Signatures of other reviewers: | Data |
