# Supplementary material for: Soy Peptide Supplementation Mitigates Undernutrition through Reprogramming Hepatic Metabolism in a Novel Undernourished Non‐Human Primate Model
Source: Adv Sci (Weinh). 2024 May 30;11(29):2306890. doi: 10.1002/advs.202306890 (PMC11304262; doi:10.1002/advs.202306890)
Supplement: Supplementary file 5 — Supporting Information Appendix 3 [file ADVS-11-2306890-s001.pdf]

ADVANCED SCIENCE
Open Access

Supporting Information

for *Adv. Sci.*, DOI 10.1002/advs.202306890

Soy Peptide Supplementation Mitigates Undernutrition through Reprogramming Hepatic Metabolism in a Novel Undernourished Non-Human Primate Model

*Zhenzhen Xu, William Kwame Amakye, Zhengyu Ren, Yongzhao Xu, Wei Liu, Congcong Gong, Chiwai Wong, Li Gao, Zikuan Zhao, Min Wang, Tao Yan, Zhiming Ye, Jun Zhong, Chuanli Hou, Miao Zhao, Can Qiu, Jieqiong Tan, Xin Xu, Guoyan Liu, Maojin Yao and Jiaoyan Ren**

aaalac
INTERNATIONAL

Achievement of Accreditation

Guangzhou Aojun Biological Technology Co., Ltd.

Guangzhou City, People's Republic of China

March 11, 2016
